# Impact of solid oxide FC-RFB and IPFC on a renewable multi-area power system using combined PI and FOPD controllers optimized by the African Vulture algorithm

**DOI:** 10.1038/s41598-025-97761-2

**Published:** 2025-05-23

**Authors:** Arindita Saha, Mahajan Sagar Bhaskar, Mahmoud F. Elmorshedy, Dhafer J. Almakhles, Sanjeevikumar Padmanaban

**Affiliations:** 1 Department of Electrical Engineering, Regent Education & Research Foundation Group of Institutions, Kolkata, 700121 West Bengal India; 2 Renewable Energy Lab, College of Engineering, Prince Sultan University, Riyadh, Saudi Arabia; 3 Electrical Power and Machines Engineering Department, Faculty of Engineering, Tanta University, Tanta, 31733 Egypt; 4 Department of Electrical Engineering, IT and Cybernetics, University of South-Eastern Norway, Porsgrunn, Norway

**Keywords:** Automatic generation control, Biodiesel, Interline power flow controller, Redox flow battery, Solid oxide fuel cell, African Vulture optimization algorithm, Cascaded controller, Electrical and electronic engineering, Energy infrastructure

## Abstract

The expanding complexity of modern energy systems and the increasing integration of renewable sources make stable load frequency control (LFC) in interconnected power networks a continuing issue. Traditional controllers, such as proportional-integral (PI), proportional-integral-derivative (PID), and other subordinate control methods, frequently fail to control frequency adequately, especially in multi-source generating systems. Furthermore, standard optimization techniques may exhibit sluggish convergence and inefficient tuning, limiting their usefulness in real-time applications. To address these problems, this study suggest an enhanced LFC framework for a three-area power system that includes thermal-biodiesel (Area-1), thermal (Area-2), and hydro-thermal (Area-3) components. The African Vulture Optimization Algorithm (AVOA) is used to improve a novel PI(FOPD) controller that combines integer-order PI with fractional-order Proportional Derivative (FOPD). According to a comparative investigation, the AVOA-augmented PI(FOPD) controller outperforms conventional I, PI, and PID controllers in terms of transient responsiveness, stability, and convergence. Additionally, AVOA outperforms optimization approaches such as Cuckoo Search, Particle Swarm Optimization, and the Firefly Algorithm. The integration of a Dish-Stirling solar thermal system, a Flexible AC Transmission System (FACTS) device, and an energy storage component improves system robustness. The results show that the AVOA-optimized PI(FOPD) controller greatly enhances LFC performance, making it a promising alternative for current power networks.

## Overview

### Inclusive outline and literature analysis of related tasks

Maintaining an efficient balance in power management is critical for guaranteeing the stability of a power system, especially when dealing with fluctuating levels of power output, demand, and losses^1–3^. This complexity becomes more obvious at times of high demand, which can result in power imbalances and system instability. Ignoring these imbalances can have major consequences, such as frequency fluctuations and inter-area power mismatches.

Automatic Generation Control (AGC)^4^ appears as an essential answer to these difficulties. It is critical to ensure system stability and dependability by dynamically altering generating levels in response to changing demand. AGC aids in maintaining the proper frequency and power balance across linked regions, reducing the dangers associated with power imbalances while improving overall system performance and reliability.

Conventional energy sources are widely used to generate power, but their constant usage accelerates resource depletion and has negative environmental effects. It is essential to switch to renewable energy sources like solar and wind to address these issues. While Acharyulu et al.^5^. studied systems integrating thermal, hydro, and gas components with renewable sources like solar, Das et al.^6^. studied integrated hybrid systems. In addition to researching thermal-gas schemes with distributed generating elements, Saha et al.^7^. concentrated on tiny hydrothermal systems with split shaft gas turbines. Babu et al.^8^. studied thermal schemes in conjunction with precision wind turbines and solar electricity, whereas Sharma et al.^9^. included solar thermal power schemes into standard AGC settings. Furthermore, research has focused on certain areas, such as the application of biodiesel in AGC (Barik et al.^10^). Research on the combination of realistic dish-Stirling solar thermal systems (RDSTS) and thermal-biodiesel-hydro schemes is still lacking, though. This suggests a chance to investigate and put into practice such a coordinated plan to improve energy efficiency and sustainability.

Energy Storage Devices (ESDs) and numerous Flexible Alternating Current Transmission System (FACTS) devices can be integrated to increase stability and minimize mistakes in a unified framework. In 2021, Mandeep et al.^11^. worked on using Interline Power Flow Controllers (IPFC). Reddy et al.^12^. covered the use of IPFC in a reorganized state, whereas Sharma et al.^13^. and Dutta et al.^14^. investigated AGC systems utilizing thyristor-controlled phase shifters.

Redox Flow Batteries (RFB)^15^, Superconducting Magnetic Energy Storage (SMES)^16^, and Ultra-capacitors (UC)^17^ are a few examples of Energy Storage Devices (ESDs) that are cited in the literature for AGC systems. Furthermore, Deng et al.^18^. presented the Solid Oxide Fuel Cell (SoFC) as an ESD; however, it has not yet been applied in AGC schemes. This offers a chance to include IPFC and SoFC^19,20^ into the standard system, improving its functionality and efficiency in regulating grid stability and power generation. Singh and Arya^21^ explored the impact of tidal turbines in microgrid frequency management in the de-loaded region, employing a unique cascade Fuzzy-FOPID droop controller. This study revealed the efficacy of using tidal turbine kinetic energy to stabilize frequency variations in standalone microgrid systems. The controller outperformed standard droop control approaches in terms of dynamic performance and robustness. Peddakapu et al.^22^ devised a control approach for regulating frequency and tie-line power in multi-microgrid systems that use intermittent renewable energy sources. They used a barnacle mating optimizer to tune a cascade CFOID-FOPIDN controller, resulting in fewer swings in frequency and tie-line power. The system, which included a variety of energy sources and storage technologies, showed better dynamic stability and robustness under changing conditions. Choudhary et al.^23^. recommended using the FOPTID + 1 controller to improve AGC performance in multi-source power systems. When combined with a global neighbourhood algorithm, it beats standard controllers in terms of frequency stabilization during load fluctuations. The addition of capacitive energy storage (CES) increased system performance even more, resulting in lower cost function values and validated stability under changing conditions.

AGC covers both primary and secondary control types, with the main goal being to choose the most appropriate subordinate controllers. There are several different types of subordinate controllers, such as dual-order subordinate controllers, cascade controllers (CaCr), fractional order (FrO)^53^, integer order (InO), and smart controllers. This range enables a sophisticated and efficient method of power system control in AGC applications. A variety of subordinate controllers are included in the array, such as fuzzy PIDF controllers^24,25^, F-TIDF-2^26^, fuzzy PI^λ^DF controllers^27^, FOPI-FOIDN setups^8^, fractional order (FrO) proportional-integral controllers, FrO-PID configurations^28^, Tilt integral Derivative (TID) controllers^13^, Cascade (CaCr) PD-PID controllers^29^, PIDN-FOPD arrangements^17^. But Saha et al..‘s^30^ use of the PI(FOPD) group is the suggested configuration that hasn’t been documented for the scheme under consideration. This gap implies a chance to add PI(FOPD) controllers to the system in order to potentially improve control performance.

Optimization algorithms or conventional approaches can be used to choose the ideal values for controller properties, which is an important step. AGC research offers a wide range of optimization strategies, but traditional approaches can be difficult and may produce worse results. They consist of the following: multiverse optimizer^11^, grasshopper optimization^31–33^, salp swarm algorithm^13^, Harris hawks optimization technique^34^, strawberry algorithm^35^, artificial flora algorithm^36^, firefly algorithm^37^, particle swarm optimization^38^, whale optimization algorithm^17^, crow search algorithm^8^, coyote optimization^27^, bird swarm algorithm^39^, hybrid moth flame pattern search optimization^40^, Hybrid Whale Optimization Algorithm^41^, grey wolf optimizer^42,52^ and arithmetic optimizer^43^. Notably, B. Abdollahzadeh et al.^44^, Quasi oppositional harmony search algorithm^51^, Genetic algorithm^54^, and Bat algorithm^55^ have developed an optimization strategy for the African Vultures Optimization Algorithm (AVOA), utilizing sound biological principles derived from vulture behaviour as their model. Further investigation and evaluation of the AVOA approach’s lack of use in AGC systems using thermal, hydro, biodiesel, and RDSTS sources is an intriguing prospect.

### Limitations derived from the literature survey

The limitations are as follows:

- Limited research on RDSTS and Hybrid Renewable Schemes: While several studies have investigated thermal, hydro, and gas-based AGC systems that incorporate renewable sources, there has been little investigation into the coupling of realistic dish-Stirling solar thermal systems (RDSTS) with thermal-biodiesel-hydro schemes. This provides an opportunity for additional research on their efficiency and sustainability.
- Integration of Solid Oxide Fuel Cells (SoFC) in AGC Systems: Although Solid Oxide Fuel Cells (SoFC) have been identified as effective energy storage devices, they have not yet been used in AGC schemes. This demonstrates a gap in their integration for improving grid stability and power generation efficiency.
- Underutilization of IPFC in AGC systems: While FACTS devices like as thyristor-controlled phase shifters have been employed in AGC, the utilization of Interline Power Flow Controllers (IPFC) in traditional AGC frameworks remains limited. Studies mostly focus on its application in a restructured state, rather than standard AGC configurations.
- The absence of PI (FOPD) controllers in AGC implementations: Despite the wide range of subordinate controllers accessible, the PI(FOPD) controller has not been documented in the AGC configurations investigated. Its incorporation could improve control performance and system stability.
- Optimization Algorithm Utilization Gaps: Several optimization techniques, including particle swarm optimization, whale optimization algorithm, and many more, have been investigated, but the African Vulture Optimization Algorithm (AVOA) has not been used in AGC systems involving thermal, hydro, biodiesel, and RDSTS sources. This provides an opportunity to assess its efficacy.
- Limited Research on Advanced Energy Storage Integration: While Redox Flow Batteries (RFB), Superconducting Magnetic Energy Storage (SMES), and Ultra-Capacitors (UC) have been studied, the overall impact of numerous energy storage technologies in AGC remains unknown.

### Main contributions of this work

The main contributions of the presented work are.

1. Investigating the PI(FOPD) Subordinate Controller with AVOA Optimization: This study is the first to integrate integer-order (InO) and fractional-order (FrO) subordinate controllers into a PI(FOPD) framework, which was optimized using the African Vulture Optimization Algorithm (AVOA). Unlike typical subordinate controllers, this architecture ensures ideal controller qualities such as greater performance, faster convergence, and improved system stability.
2. Triple-Arena System Configuration: A three-region AGC model is considered:
  Arena-1: Thermal and biodiesel power generation using FACTS devices (IPFC) and renewable energy (RDSTS).
  Arena-2 generates entirely through thermal energy.
  Arena-3: Hydrothermal combination adds a new dimension to current AGC research.
  This arrangement integrates many energy sources to increase system stability and performance.
3. Integration of Advanced Energy Storage Technology: This paper discuss a complete energy storage architecture that includes Redox Flow Batteries (RFB), Solid Oxide Fuel Cells (SoFC), and other energy storage technologies. Previous studies had not considered the incorporation of SoFC along with RFB in AGC, hence this represents a significant improvement in power system stability research.
4. Consideration of Generation Rate Constraints (GRC): Unlike traditional AGC models, this work uses GRC for multiple system components, making the study more realistic and applicable to real-world scenarios.
5. Performance Evaluation with ISE: The PI(FOPD) subordinate controller is carefully evaluated using system constraints, including Integral Squared Error (ISE) as a performance metric, to ensure a quantitative measure of efficiency and resilience.

### Purposes of current pieces of writing

The present written works aim to achieve the following main goals:

I. To create a triple-arena structure in the plan, with thermal and biodiesel components located in arena-1, thermal components in arena-2, and hydrothermal components in arena-3.
II. To evaluate the dynamics of the scheme with different controllers, such I, PI, PID, and PI(FOPD), and to use the AVOA to determine which controller works best.
III. Assessing the scheme’s effectiveness with the outstanding subordinate controller and utilizing a variety of algorithms, including CS, FA, PSO, and AVOA, to choose the best optimization technique.
IV. To evaluate the scheme’s effectiveness when dish-stirling solar thermal components are realistically present.
V. To assess the scheme’s effectiveness when the Interline Power Flow Controller (IPFC) is included.
VI. To evaluate the scheme’s effectiveness while using a Solid Oxide Fuel Cell (SoFC) in conjunction with a Redox Flow Battery (RFB).

### Assemblage of current work

The present composition is structured into many sections:

1. Introduction: An outline and background of the subject are given in this part.
2. Scheme Under Investigation: The particular scheme under investigation is described in this section.
3. Commended Subordinate Controller: The suggested subordinate controller is covered in this section.
4. Enforced Optimization Method: The optimization strategy used for the study is described in this section.
5. Conclusion and Assessment: The findings and their assessment are given in this section.
6. Review of the Entire Task: This part provides a thorough analysis of the task.

## Scheme being assessed

An analysis is conducted on a three-region system with different kinds and a region size ratio of 1:3:4. The system consists of hydro and thermal units in Region 3, thermal and biodiesel units in Region 1, and thermal units in Region 2. Individual generating units in each region have the following participation factors (ppf): ppf_11_ = 0.61, ppf_12_ = 0.39 for Region 1, ppf_21_ = 0.49, ppf_22_ = 0.51 for Region 2, and ppf_31_ = 0.46, ppf_32_ = 0.54 for region 3. This arrangement is known as Scheme A.

Scheme B is then created by combining Scheme A with a practical dish-stirling solar thermal system (RDSTS). In Scheme B, the following criteria determine participation: in region 1, ppf_11_ = 0.3, ppf_12_ = 0.39, ppf_13_ = 0.31; in region 2, ppf_21_ = 0.21, ppf_22_ = 0.41, ppf_23_ = 0.38; and region 3, ppf_31_ = 0.25, ppf_32_ = 0.41, ppf_33_ = 0.34.

Scheme B is then combined with a FACTS mechanism, especially an interline power flow controller (IPFC), to produce Scheme C. Scheme D is then formed by the integration of energy storage components, namely the redox flow battery (RFB) and solid oxide fuel cell (SoFC).

**Fig. 1:**
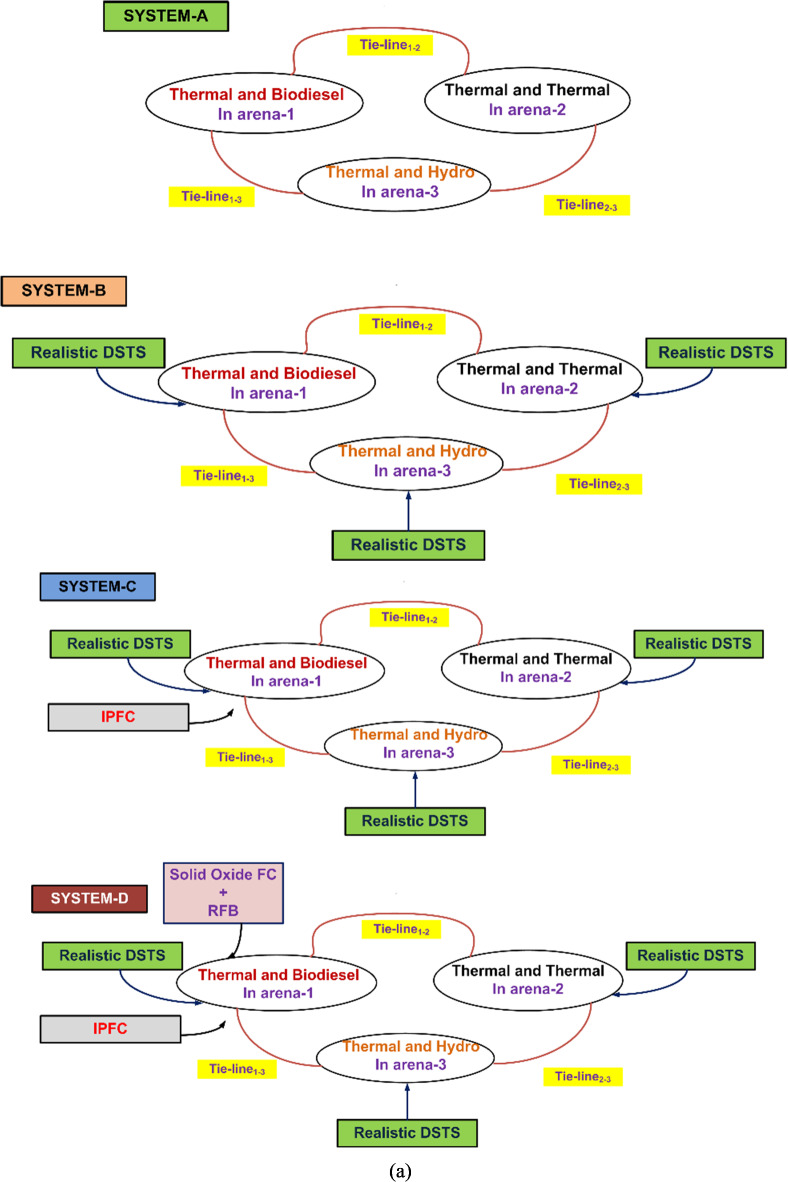

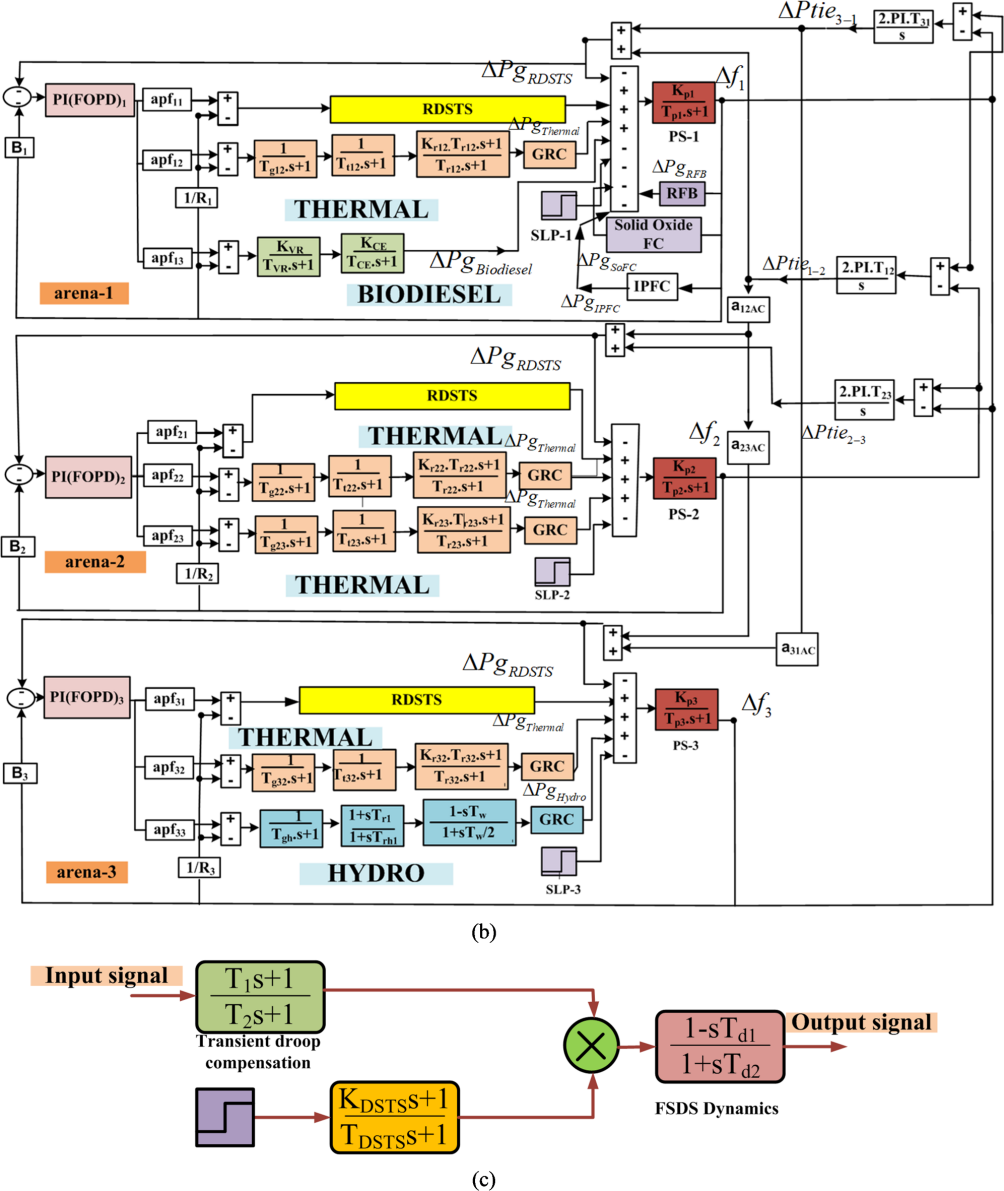
The considered scheme’s assembly and transfer function prototype with RDSTS, IPFC, solid oxide FC, and RFB present (**a**) putting the considered scheme together (**b**) the considered scheme’s transfer function prototype with RDSTS, IPFC, solid oxide FC, and RFB present (**c**) RDSTS transfer function prototype^45^.

Figure 1a and b show the representation and transfer function (Trfn) models of these schemes, respectively. The African Vultures Optimization Algorithm (AVOA) is used to determine the optimal values of each subordinate controller parameter and associated restrictions while taking the integral squared error (*Pi*_*ISE*_) performance index, which is given by Eq. (1)^16^.

1 $$P{i_{ISE}}=\int\limits_{0}^{T} {\left\{ {{{\left( {\Delta {f_1}} \right)}^2}+{{\left( {\Delta {f_2}} \right)}^2}+{{\left( {\Delta {f_3}} \right)}^2}+{{\left( {\Delta {P_{ti{e_{1 - 2}}}}} \right)}^2}+{{\left( {\Delta {P_{ti{e_{2 - 3}}}}} \right)}^2}+{{\left( {\Delta {P_{ti{e_{1 - 3}}}}} \right)}^2}} \right\}{\text{ }}d} t$$

### Bio-diesel component

A biodiesel-generating unit is presently taking the place of a normal diesel-producing unit since biodiesel is less viscous, non-hazardous, and biodegradable. It is also considerably more compact. It is a good substitute for producing power since it also releases less carbon monoxide. An ignition engine and a valve controller make up the biodiesel-producing unit. Equations (2) and (3), respectively, reflect the first-order transfer functions (Trfn) of the biodiesel-producing unit’s valve controller and ignition engine^10^.

2 $$\:{Trfn}_{valve\:regulator\:(bio-diesel)}=\frac{{K}_{valve\:regulator}}{1+s{T}_{valve\:regulator}}$$

The valve controller’s gain and time factors are denoted by *K*_*valve regulator*_ and *T*_*valve regulator*_, respectively.

3 $$\:{Trfn}_{combustion\:engine\:(bio-diesel)}=\frac{{K}_{combustion\:engine}}{1+s{T}_{combustion\:engine}}$$

The ignition engine’s gain and time factors are denoted as *K*_*combustion engine*_ and *T*_*combustion engine*_, respectively.

### RDSTS-realistic dish-stirling solar thermal system

A parabolic dish, a tracking device, and a receiver make up a Dish-Stirling Solar Thermal System (DSTS). All of the heat energy that the parabolic dish receives is reflected and focused on the receiver. The working fluids are heated by the concentrated energy, and the Stirling engine is hence powered. By converting thermal energy into rotational energy, this engine generates electrical energy via driving the squirrel-cage induction generator (SCIG). Equation (4) represents the DSTS’s transfer function (Trfn)^45^.

4 $$\:{Trfn}_{Dish-Stirling\:STS}=\frac{{K}_{Dish-Stirling\:STS}}{1+s{T}_{Dish-Stirling\:STS}}$$

The gain and time factors of the DSTS are denoted as *K*_*Dish Stirling STS*_ and *T*_*Dish Stirling STS*_, respectively.

Due to the intrinsic speed characteristics of SCIGs, Squirrel-Cage Induction Generators (SCIGs) are unable to maintain frequency requirements because of changes in generator speed. Therefore, SCIGs have a transient-droop property for effective functioning. The delivery of solar energy to the grid must also be restricted in order to avoid the introduction of instability brought on by the DSTS system’s non-minimum phase nature. As seen in Fig. 1(c), this results in the installation of a Realistic Dish-Stirling Solar Thermal System (RDSTS) with non-minimum phase and transient droop characteristics. Equation (5) provides the RDSTS’s transfer function (Trfn) model^45^.

5 $$\:{Trfn}_{Realistic\:DSTS}=\frac{1}{s}\times\:\left(\frac{{K}_{Dish\:Stirling\:STS,\:i}}{1+s{T}_{Dish\:Stirling\:STS,\:i}}\right)\times\:\left(\frac{{T}_{i1}s+1}{{T}_{i2}s+1}\right)\times\:\left(\frac{-{T}_{di1}s+1}{{T}_{di2}s+1}\right)$$

### Energy storage device: redox flow battery (RFB)

One kind of flow battery that is based on static energy storage techniques is the Redox Flow Battery (RFB). Applications for RFBs in Automatic Generation Control (AGC) are many. In contrast to conventional batteries, an RFB stores its reactive material in external tanks rather than inside the battery construction itself. Therefore, the amount of electrolyte in these external storage reservoirs determines the overall energy capacity, whereas the electrode design affects power output. Usually, a combination of vanadium ions and sulfuric acid makes up the electrolyte.

The electrolyte solution is pumped between two pumps and the battery’s electrochemical cells. Equations (6) and (7), respectively, depict the electrochemical processes that take place inside the battery’s electrochemical cells during charging and discharging^15^.

At the positive electrode’s location:

6 $${V^{4+}}\underset{{Disch\arg e}}{\overset{{Ch\arg e}}{\longleftrightarrow}}{V^{5+}}+{e^ - }$$

At the negative electrode’s location:

7 $${V^{3+}}+{e^ - }\underset{{Disch\arg e}}{\overset{{Ch\arg e}}{\longleftrightarrow}}{V^{2+}}$$

The Redox Flow Battery (RFB) is distinguished by its long operating life and high power production capability. It has a number of benefits, such as high efficiency, immunity to self-discharge problems, rapid and temporary increased capacity, cost-effectiveness, and robustness against unanticipated fluctuations or disruptions.

### The SoFC, or solid oxide fuel cell

According to Deng et al.^18^, the Solid Oxide Fuel Cell (Solid Oxide FC) is a stationary electrochemical device that transforms hydrogen and oxygen’s chemical energy into electrical energy. Porous electrodes and an electrolyte of the ceramic kind are used to accomplish this. The fuel processing unit, power generation unit, and power conditioning unit are the three primary parts involved in producing power from Solid Oxide FC.

High operating temperatures, high efficiency, robust catalytic reactions, minimal susceptibility to degradation, efficacy, and decreased exhaust gas emissions are just a few of the benefits that Solid Oxide FC offers. Equation (8) represents the Solid Oxide FC ‘s transfer function (Trfn)^18^.

8 $$\:{Trfn}_{Solid\:Oxide\:FC}=\frac{{K}_{Solid\:Oxide\:FC}}{1+s{T}_{Solid\:Oxide\:FC}}$$

*K*_*Solid-oxide FC*_ and *T*_*Solid-oxide FC*_ are the gain and time factors, respectively, of the Solid Oxide Fuel Cell (Solid Oxide FC).

### Advised controller-PI (FOPD)

A fractional order (FrO) controller and an integer order (InO) controller are combined in the controller, PI(FOPD). In particular, it combines a FrO proportional-derivative (FOPD) controller with an InO proportional-integral (PI) controller. The purpose of this setup is to improve system performance by utilizing the advantages of both kinds of controllers.

**Fig. 2:**
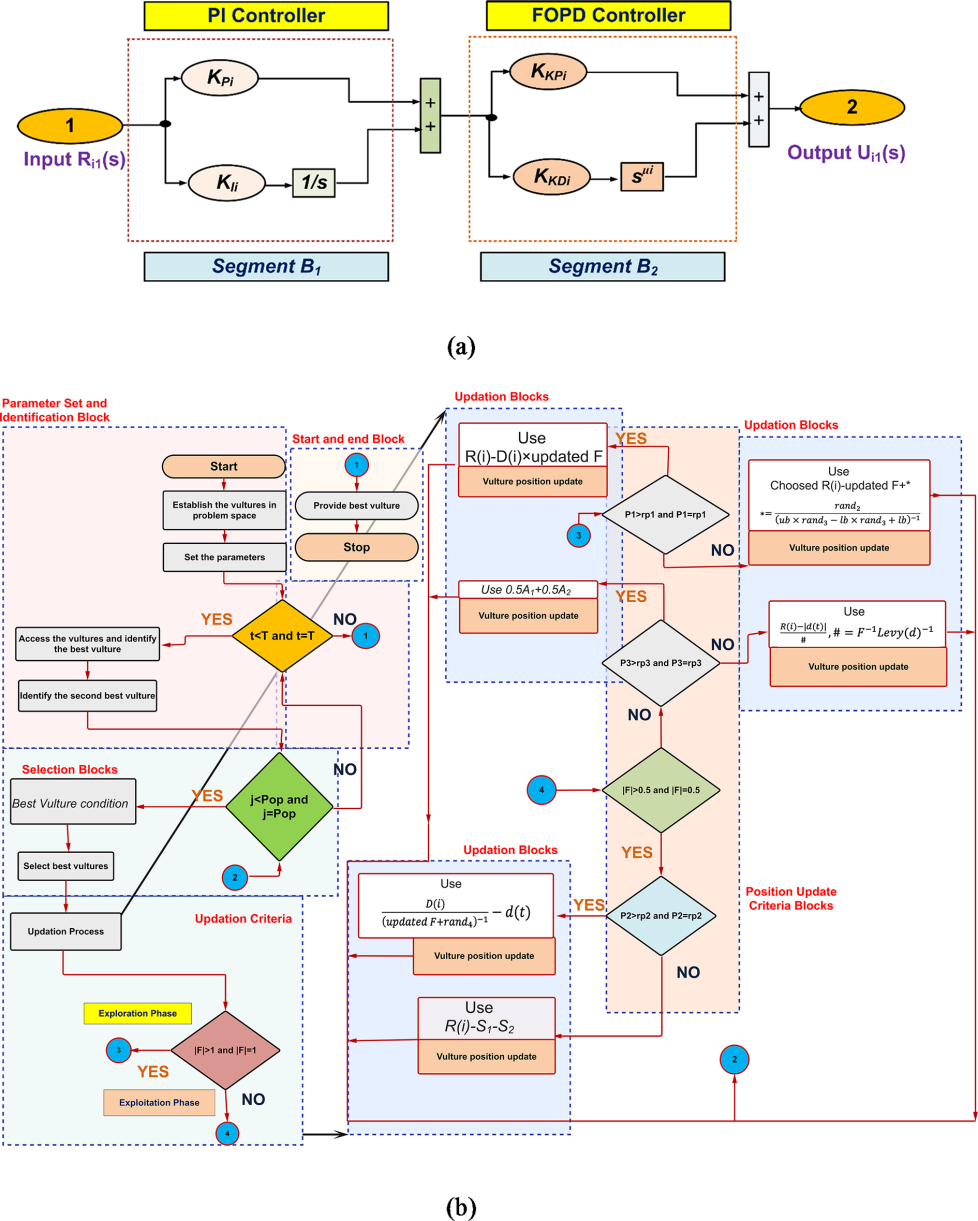
Construction of the recommended controller and flow chart: (**a**) the commended PI (FOPD) controller assembly^30^, (**b**) Flow diagram of AVOA method^44^.

Setting up the PI (FOPD): The PI(FOPD) controller’s setup is shown in Fig. 2(a): The PI controller is represented by Segment 1 (*B1*). The FOPD controller is represented by Segment 2 (*B2*). In the controller for PI(FOPD): The output signal is *O*_*si*_(s). The reference signal is *R*_*si*_(s). Equation (9) gives the transfer function of the PI controller segment^30^.

9 $$\:{Trfn}_{{PI}_{ith\_area}}={K}_{{P}_{ith\_area}}+\raisebox{1ex}{${K}_{{I}_{ith\_area}}$}\!\left/\:\!\raisebox{-1ex}{$s$}\right.$$

For *i*^*th*^ recommended area, the integral gain is represented as *K*_*Iith_area*_ and the proportional gain as *K*_*Pith_area*_.

The mathematical link between the input and output signals for the PI component is captured by Eq. (9), which represents the transfer function for the PI controller segment (*B*_*1*_).

The FrO derivative’s Riemann-Liouville summation may be obtained from (10)^30^. The notion of an integer-order derivative is extended to non-integer orders by the Riemann-Liouville fractional order (FrO) derivative, which offers a wider definition of differentiation. This type of differentiation, which can perform better than conventional integer-order derivatives in a variety of applications, including control systems, is crucial to fractional calculus.

10 $$\:\alpha\:{D}_{t}^{\alpha\:}f\left(t\right)=\frac{1}{{\Gamma\:}\left(n-\alpha\:\right)}\frac{{d}^{n}}{{dt}^{n}}{\int\:}_{\alpha\:}^{t}{\left(t-\tau\:\right)}^{n-\alpha\:-1}f\left(\tau\:\right)d\tau\:,\:n-1<n,\:n\:ia\:an\:integer$$

Γ(.) is the Euler’s gamma function, $\alpha \mathop D\nolimits_{t}^{\alpha }$ is the fractional operator. Equation (11) provides the Fro derivative modification in the Laplace domain^30^.

11 $$\:L\left\{\alpha\:{D}_{t}^{\alpha\:}f\left(t\right)\right\}={S}^{\alpha\:}F\left(s\right)-\sum\:_{k=0}^{n-1}{S}^{k}\alpha\:{D}_{t}^{\alpha\:-k-1}f\left(t\right){|}_{t=0}$$

Oustaloup et al.^46^. show the drawbacks of computing poles and zeros infinitely because of their absolute likeness. Here, a useful Trfn is discussed, which may be used to approximate FrO derivatives together with integrators using the recursive distribution around poles and zeros, as shown by (12)^46^.

12 $$\:{s}^{\alpha\:}=K\prod\:_{n=1}^{M}\frac{1+\left(\raisebox{1ex}{$s$}\!\left/\:\!\raisebox{-1ex}{${\omega\:}_{Z,n}$}\right.\right)}{1+\left(\raisebox{1ex}{$s$}\!\left/\:\!\raisebox{-1ex}{${\omega\:}_{p,n}$}\right.\right)}$$

Assume that the tuned gain *K = 1*, gain = 0 dB through 1 rad/s frequency, *M* = The predetermined count of poles and zeros, and that the frequencies selected for the poles and zeros are represented by (13)–(17)^46^.

13 $$\:{\omega\:}_{Z,l}={\omega\:}_{l}\sqrt{n}$$

14 $$\:{\omega\:}_{p,n}={\omega\:}_{Z,n}\epsilon\:,\:\:\:\:\:\:\:\:\:\:\:\:\:\:\:\:\:\:\:\:\:\:\:\:\:\:\:\:\:\:\:\:\:\:\:\:\:\:\:\:n=1,\dots\:\dots\:,M$$

15 $$\:{\omega\:}_{Z,\:n+1}={\omega\:}_{p,n}\sqrt{\eta\:}$$

16 $$\:\epsilon\:={\left(\raisebox{1ex}{${\omega\:}_{h}$}\!\left/\:\!\raisebox{-1ex}{${\omega\:}_{l}$}\right.\right)}^{\raisebox{1ex}{$\nu\:$}\!\left/\:\!\raisebox{-1ex}{$M$}\right.}$$

17 $$\:\eta\:={\left(\raisebox{1ex}{${\omega\:}_{n}$}\!\left/\:\!\raisebox{-1ex}{${\omega\:}_{l}$}\right.\right)}^{\raisebox{1ex}{$(1-v)$}\!\left/\:\!\raisebox{-1ex}{$M$}\right.}$$

The FOPD Trfn is provided by (18)^30^.

18 $$\:{Trfn}_{\left(FOPD\right)i}={\left({K}_{KP}\right)}_{i}+{\left({K}_{KD}\right)}_{i}{s}^{{\mu\:}_{i}}$$

Equation (19) reveals the Trfn for the PI(FOPD) subordinate controller^30^.

19 $$\:{Trfn}_{PI\left(FOPD\right)i}=\left({K}_{Pi}+\raisebox{1ex}{${K}_{Ii}$}\!\left/\:\!\raisebox{-1ex}{$s$}\right.\right)\times\:{\left({K}_{KP}\right)}_{i}+{\left({K}_{KD}\right)}_{i}{s}^{{\mu\:}_{i}}$$

Figure 2(a) shows the Trfn example of the dual stage PI(FOPD) subordinate controller. AVOA boosts the parameters linked to the subordinate controller’s recommended PI(FOPD) with margins in (20)^30^.

20 $$\:\begin{array}{c}0\le\:{K}_{Pi}\le\:1,\:0\le\:{K}_{Ii}\le\:1\\\:0\le\:{K}_{KPi}\le\:1,\:0\le\:{K}_{KDi}\le\:1\:and\:0\le\:{\mu\:}_{i}\le\:1\end{array}$$

### African vultures optimization algorithm (AVOA)

Abdollahzadeh and associates^44^ developed the optimization method for African vultures optimization algorithm (AVOA). This algorithm incorporates strong biological principles into its base by taking cues from vulture behaviour and robust biological principles. The AVOA by default is shown as follows:

- Vultures may or may not be present in a certain setting, with a maximum of N vultures possible. According to this number, metaheuristic algorithms choose a population size, and the precise count is determined by the type of issue that researchers want to solve with the AVOA.
- A large number of vultures in the wild can be divided into two different categories. To divide the vultures into these groups, the algorithm first determines the fitness function for each solution in the original population. The algorithm chooses the primary and top-ranked vulture as the one with the best response, and the second vulture as the one with the second-best response. In each iteration, the other options comprise a population that is capable of supplanting or replacing one of the two top-performing vultures.
- This algorithm’s logic for grouping vultures is based on one of their core natural behaviours: cooperative group scavenging to find and access food supplies. There are differences between each group of vultures’ capacity to locate and eat food.
- Vultures are naturally inclined to persistently scavenge and conduct extended searches for food, which prevents them from becoming trapped by hunger. We assume that the weakest and most famished solution within the population reflects the worst-case situation throughout the formulation phase of our anti-hunger strategy. As a result, the vultures work to keep a distance from this less-than-ideal option and to converge on the ideal one. The two strongest and most skilled vultures in the AVOA framework are selected as the two best solutions, and the other vultures make an effort to approach these top performers.

The Artificial Vulture’s Optimization Algorithm (AVOA) was developed through a methodical formulation process separated into four different parts, building upon the core principles of vultures and combining four crucial assumptions. Figure 2(b) provides a visual depiction of the AVOA’s flowchart and pseudo-code of AVOA is as follows:

**Algorithm:**
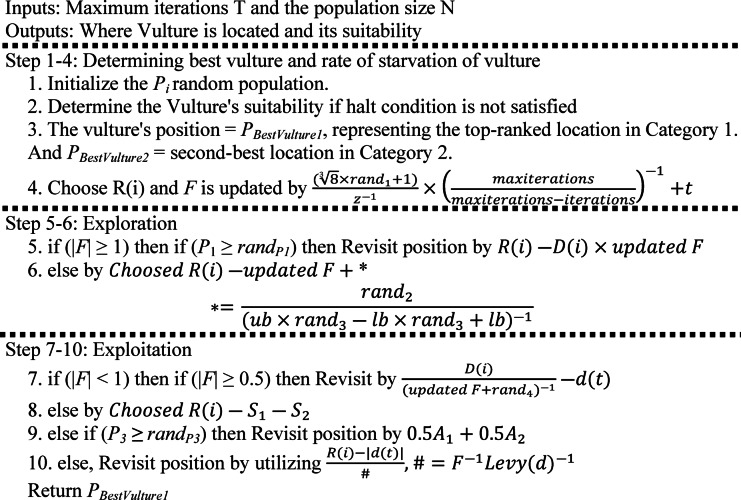
AVOA.

## The results and assessment

### Assessing the possible results to identify the ideal controller element

**Fig. 3:**
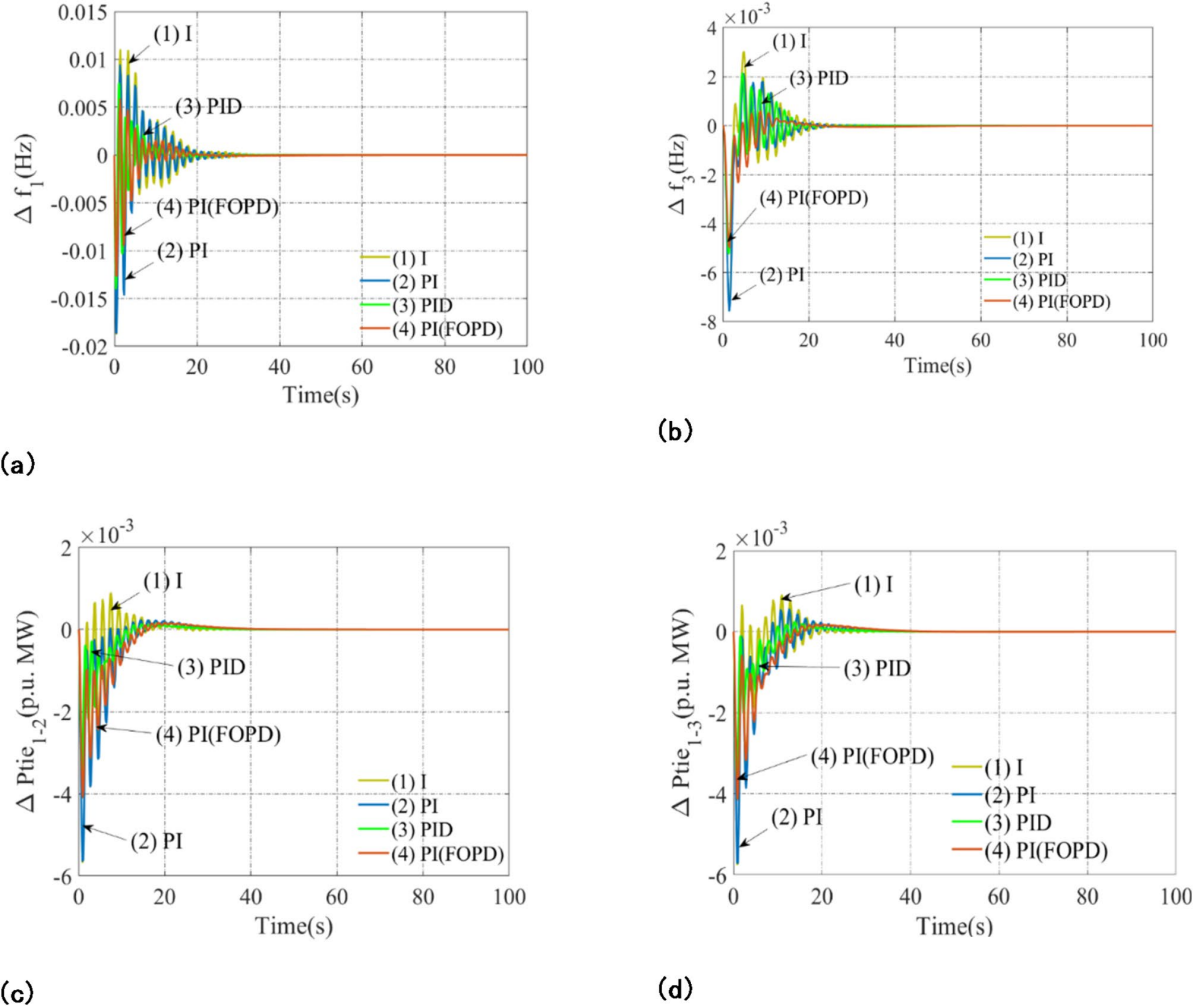
The following describes the evaluation of controller results for scheme-A, with a particular focus on a 1% step load disturbance: (**a**) Arena-1 Frequency Anomaly, (**b**) Arena-3 Frequency Anomaly, (**c**) Line Interlinking Arena-1-2 Tie Line Power Anomaly, (**d**) Line Interlinking Arena-1-3: Tie Line Power Anomaly.

Thermal-biodiesel components are positioned in Arena-1, thermal elements in Arena-2, and thermal-hydro elements in Arena-3 (Scheme-A), resulting in a diverse multi-area power system aimed to improve power generation and distribution stability and efficiency. In this architecture, several subordinate controllers are built individually, including I, PI, PID, and PI(FOPD). A 1% step load disturbance in arena-1 is applied to the system as part of the assessment procedure. The African Vulture Optimization Algorithm (AVOA) method is used to find the optimal values of each subordinate controller parameter and associated restrictions. The Integral Squared Error (*J*_*ISE*_) serves as the objective function. Table 1 displays these ideal values, whereas Fig. 3 shows the relevant reactions. A thorough analysis of the results highlights the PI(FOPD) controller’s superiority over the other subordinate controllers in terms of decreased peak deviations (Peak_O, Peak_U), fluctuation amplitude, and settling time (Set_Time). The effectiveness of several controllers—integral (I), proportional-integral (PI), proportional-integral-derivative (PID), and fractional-order proportional-integral-derivative (PI(FOPD))—in managing frequency and tie-line power deviations is evaluated in the Table 2. For each characteristic, peak overshoot (Peak_O), peak undershoot (Peak_U), and settling time (set-time) are used to assess performance.

The PI(FOPD) controller exhibits the lowest peak overshoot (0.0049) and peak undershoot (0.0127) for frequency deviation (Δ*f*_1_), suggesting superior stability and control. Furthermore, it has the quickest settling time—32.27 s—while the Integral controller responds the slowest—36.98 s. Likewise, the PI(FO-PD) controller has exceptional performance for Δ*f*_3_ with the quickest settling time of 28.54 s and the fewest peak deviations. Although it operates admirably as well, the PID controller is marginally inferior. PI(FOPD) still performs better than other controllers for tie-line power fluctuations. The Integral controller takes the longest time, at 35.23 s, while the Δ*Ptie*_*1−2*_ has the fastest settling time of 31.43 s and the lowest peak deviations. In a similar way, PI(FOPD) yields the best results for Δ*Ptie*_*1−3*_ with a settling time of 35.12 s, whereas the Integral controller takes 37.11 s.

Overall, the PI(FOPD) controller emerges as the best choice for system stability, offering the lowest peak deviations and the fastest response across all characteristics.

**Table 1:** Finest values of subordinate controllers for scheme-A.

Controllers	Constraints/gains	Arena-1	Arena-2	Arena-3
I	K_Ii_*	0.9864	0.8705	0.9177
PI	K_Pi_*	0.0966	0.0578	0.0647
	K_Ii_*	0.7076	0.7289	0.6898
PID	K_Pi_*	0.9763	0.6619	0.9715
	K_Ii_*	0.9883	0.9778	0.9791
	K_Di_*	0.4239	0.4788	0.4793
PI(FOPD)	K_Pi_*	0.0814	0.0532	0.0712
	K_Ii_*	0.3689	0.1471	0.1567
	K_FPi_*	0.0876	0.0685	0.0814
	K_FDi_*	0.8799	0.8891	0.8719
	µ_i_*	0.0091	0.0088	0.0061

**Table 2:** Assessment of effective outcomes concerning varied characteristics employing different controllers.

Characteristics	I	PI	PID	PI(FOPD)
***Δf***_***1***_				
Peak_O	0.0123	0.0087	0.0061	0.0049
Peak_U	0.0187	0.0186	0.0144	0.0127
Set_Time	36.98	35.67	34.34	32.27
***Δf***_***3***_				
Peak_O	0.0031	0.0022	0.0021	0.0001
Peak_U	0.0078	0.0077	0.0055	0.0051
Set_Time	32.56	30.34	29.11	28.54
***ΔPtie***_***1-2***_				
Peak_O	0.0097	0.00013	0.00012	0.00011
Peak_U	0.0058	0.0057	0.0056	0.0041
Set_Time	35.23	34.22	33.34	31.43
***ΔPtie***_***1-3***_				
Peak_O	0.0012	0.00016	0.00013	0.00012
Peak_U	0.0058	0.0057	0.0056	0.0042
Set_Time	37.11	36.87	36.34	35.12

### Thorough comprehension of the impact of various algorithms performance using the PI(FOPD) controller

**Fig. 4:**
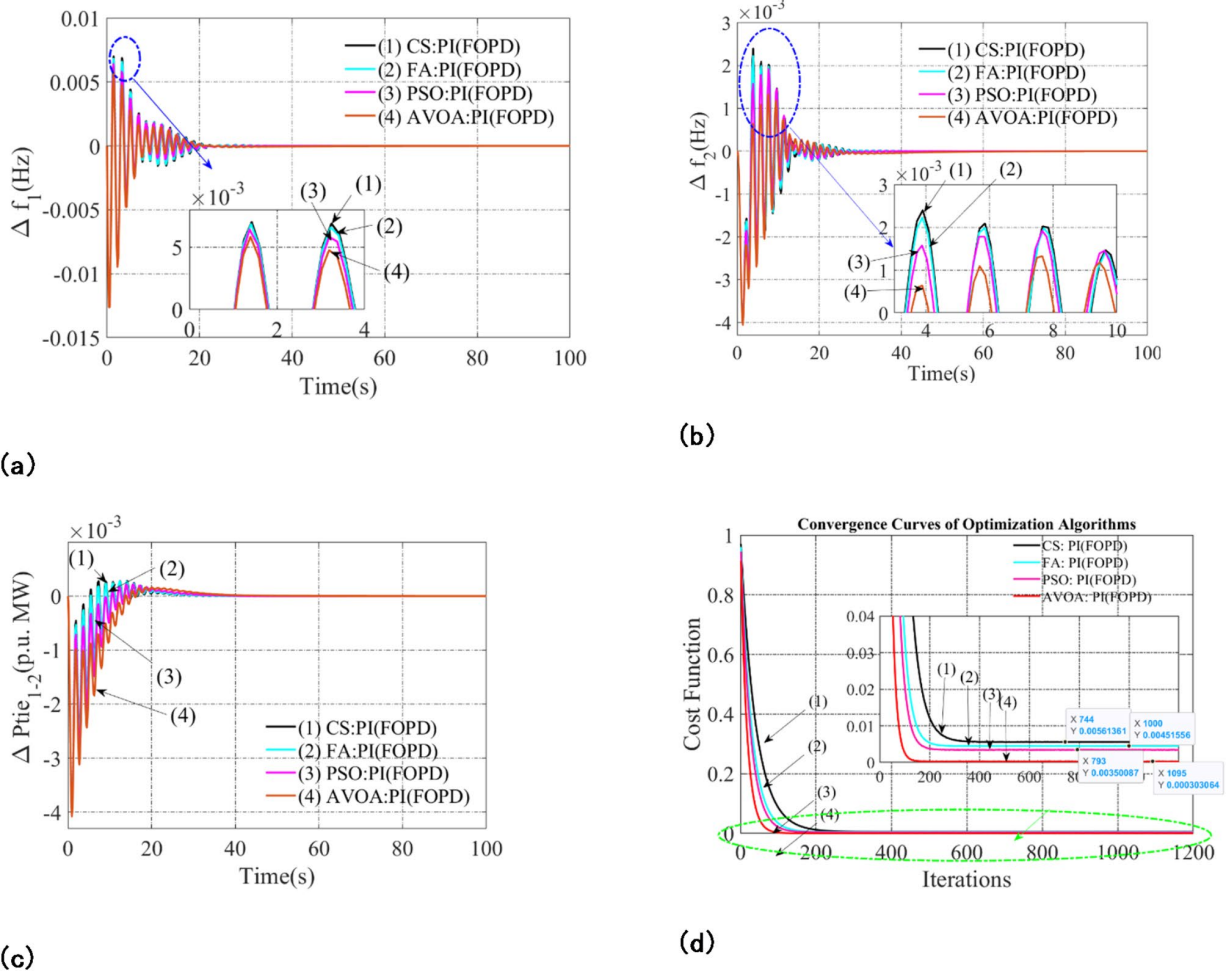
Examining the appropriateness of algorithms like AVOA, PSO, CS, and FA for optimizing the PI(FOPD) subordinate controller’s parameters in scheme-A: (**a**) Frequency anomalies in Arena-1, (**b**) Frequency anomalies in Arena-2, (**c**) Tie line power anomalies for line interlinking Arena-1-2, (**d**) Contrasting Convergence Curvature.

It is observed that the PI(FOPD) subordinate controller is the most efficient one in the previous section. Here, techniques like African Vulture Optimization Algorithm (AVOA), Firefly Algorithm (FA), Particle Swarm Optimization (PSO), and Cuckoo Search (CS) to further improve the PI(FOPD) subordinate controller. Table 3 compiles the optimal parameter values and associated restrictions that emerge from CS, FA, and PSO. Figure 4a–c show the results of each method. Furthermore, Fig. 4(d) contrasts the convergence curves.

A thorough examination of Fig. 4a–d demonstrates that the AVOA-enhanced controller performs better. A closer look at the controller parameters acquired by AVOA reveals lower values of Peak_O, Peak_U, and Set_Time. Moreover, the divergent convergence curves shown in Fig. 4(d).

Based on important performance metrics, Table 4 compares and contrasts several optimization methods, including the African Vulture Optimization Algorithm (AVOA), Firefly Algorithm (FA), Particle Swarm Optimization (PSO), and Cuckoo Search (CS). These consist of integral squared error (PI_ISE_), settling time (set-time), peak overshoot (Peak_O), and peak undershoot (Peak_U). Frequency deviations (Δ*f*_1_, Δ*f*_2_) and tie-line power deviation (Δ*Ptie*_1−2_), which are crucial markers of system stability and dynamic performance, are taken into account when making the assessment. With the lowest peak overshoot (0.0049) of any method, AVOA exhibits superior transient response control in terms of frequency deviation (Δ*f*_1_). Furthermore, compared to previous approaches, the peak undershoot (0.0127) is still somewhat lower. Above all, the fastest settling time (32.27 s) is achieved by AVOA, suggesting a quicker return to steady-state conditions and fewer oscillations. AVOA performs better than other optimization methods for frequency deviation (Δ*f*_2_). With the lowest peak overshoot (0.0006), it exhibits improved damping properties. In addition, the peak undershoot (0.0040) is marginally better than with previous methods. Additionally, AVOA has a substantially shorter settling time (35.88 s), demonstrating how well it works to enhance system stability. When it comes to assessing tie-line power deviation (ΔPtie _1−2_,), AVOA offers significant gains. Better control over power fluctuations is ensured by the strategy with the lowest peak overshoot (0.00011). With slight modifications, the peak undershoot (0.0041) is still competitive. Furthermore, AVOA exhibits the potential to reduce oscillations in power exchange between related areas by achieving the fastest settling time (31.43s).

Finally, AVOA is used to minimize the integral squared error (PI_ISE_), which indicates the entire system variation over time. AVOA outperforms other algorithms with an error value of 0.00303, demonstrating its capacity to increase system resilience, reduce frequency deviations, and boost overall control effectiveness.

The findings show that AVOA continuously performs better than CS, FA, and PSO in every parameter that was tested. Its advantage is demonstrated by its reduced integral squared error, faster settling time, and lower peak overshoot and undershoot. These results highlight AVOA’s potential to increase power system stability, lessen fluctuations, and guarantee a more dependable and effective energy management approach.

**Table 3:** Related gains and constraints of PI(FOPD) subordinate controller employing assorted procedure for scheme-A.

CS	K_P1_ = 0.0876	K_I1_ = 0.6663	K_KP1_ = 0.0875	K_KD1_ = 0.9775
	µ_1_ = 0.0077	K_P2_ = 0.0685	K_I2_ = 0.5841	K_KP2_ = 0.0594
	K_KD2_ = 0.9868	µ_2_ = 0.0079	K_P3_ = 0.0795	K_I3_ = 0.6656
	K_KP3_ = 0.0886	K_KD3_ = 0.0977	µ_3_ = 0.0086	
FA	K_P1_ = 0.0874	K_I1_ = 0.6734	K_KP1_ = 0.0675	K_KD1_ = 0.9475
	µ_1_ = 0.0055	K_P2_ = 0.0863	K_I2_ = 0.6662	K_KP2_ = 0.0723
	K_KD2_ = 0.9475	µ_2_ = 0.0042	K_P3_ = 0.0846	K_I3_ = 0.7632
	K_KP3_ = 0.0575	K_KD3_ = 0.8556	µ_3_ = 0.0064	
PSO	K_P1_ = 0.0873	K_I1_ = 0.5638	K_KP1_ = 0.0885	K_KD1_ = 0.8865
	µ_1_ = 0.0077	K_P2_ = 0.0676	K_I2_ = 0.4323	K_KP2_ = 0.0758
	K_KD2_ = 0.8786	µ_2_ = 0.0076	K_P3_ = 0.0778	K_I3_ = 0.4556
	K_KP3(_ = 0.0998	K_KD3_ = 0.8876	µ_3_ = 0.0075	

**Table 4:** Assessment of effective outcomes with reference to varied characteristics employing assorted algorithms.

Characteristics	CS	FA	PSO	AVOA
***Δf***_***1***_				
Peak_O	0.0069	0.0066	0.0059	0.0049
Peak_U	0.0128	0.0128	0.0128	0.0127
Set_Time	35.25	34.50	33.54	32.27
***Δf***_***2***_				
Peak_O	0.0026	0.0024	0.0017	0.0006
Peak_U	0.0042	0.0041	0.0042	0.0040
Set_Time	43.14	39.93	37.33	35.88
***ΔPtie***_***1-2***_				
Peak_O	0.00024	0.00023	0.00022	0.00011
Peak_U	0.0044	0.0043	0.0042	0.0041
Set_Time	37.12	36.67	35.54	31.43
***Pi***_***ISE***_	0.00561	0.00451	0.00351	0.00303

### Assessing the possible results to identify the ideal performance index

**Fig. 5:**
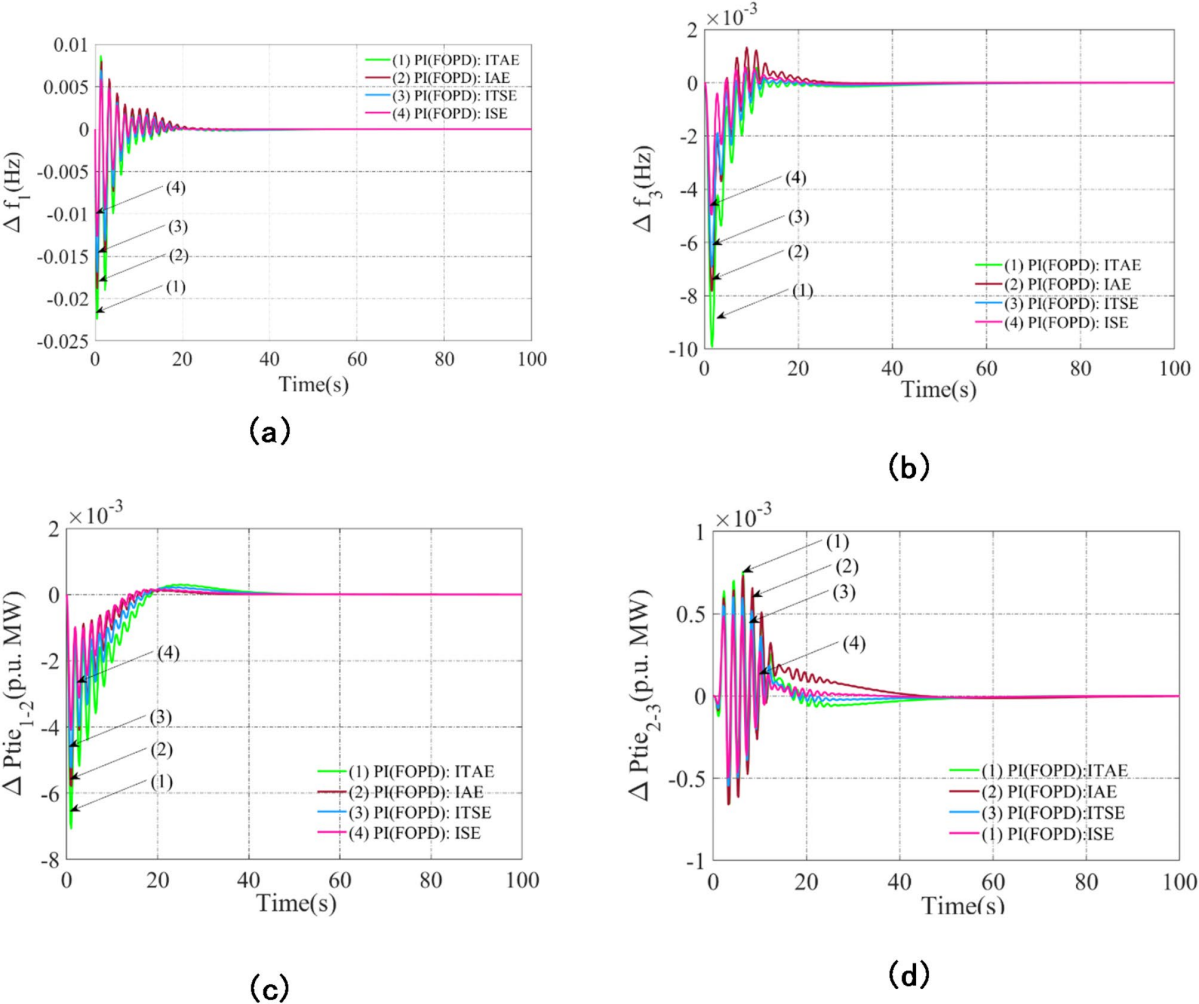
Examining the appropriateness of performance indices like ITAE, IAE, ITSE, and ISE for optimizing the PI(FOPD) subordinate controller’s parameters in system: (**a**) Frequency anomalies in Arena-1, (**b**) Frequency anomalies in Arena-3, (**c**) Tie line power anomalies for line interlinking Arena-1-2, (**d**) Tie line power anomalies for line interlinking Arena-2-3.

The basic performance index (*Pi*) for integral squared error (*Pi*_*ISE*_), integral time squared error (*Pi*_*ITSE*_), integral absolute error (*Pi*_*IAE*_), and integral time absolute error (*Pi*_*ITAE*_) is derived by assessing the system with each index separately using the PI(FOPD) controller. The AVOA method determines the appropriate gains and parameters for the PI(FOPD) controller. Equations (1), (21)-(23) provide mathematical expressions for *Pi*_*ISE*_, *Pi*_*ITSE*_, *Pi*_*IAE*_, and *Pi*_*ITAE*_^47^. Figure 5a–d compare the dynamic responses using the optimal PI(FOPD) controller parameters for each *Pi*. A thorough examination of these answers reveals that the system performs better when *Pi*_*ISE*_ is used as the performance index, resulting in lower Peak_O, Peak_U, and set-time. Furthermore, the computed values of the performance indices are: *Pi*_*ISE*_ = 0.00303, *Pi*_*ITSE*_ = 0.0044, *Pi*_*IAE*_ = 0.0051, and *Pi*_*ITAE*_ = 0.0057, indicating that *Pi*_*ISE*_ delivers the best overall system performance.

21 $$P{i_{ITSE}}=\int\limits_{0}^{T} {\left\{ {{{\left( {\Delta {f_1}} \right)}^2}+{{\left( {\Delta {f_2}} \right)}^2}+{{\left( {\Delta {f_3}} \right)}^2}+{{\left( {\Delta {P_{ti{e_{1 - 2}}}}} \right)}^2}+{{\left( {\Delta {P_{ti{e_{2 - 3}}}}} \right)}^2}+{{\left( {\Delta {P_{ti{e_{1 - 3}}}}} \right)}^2}} \right\} \times t{\text{ }}d} t$$

22 $$P{i_{IAE}}=\int\limits_{0}^{T} {\left\{ {\left| {\Delta {f_1}{\text{ }}} \right|+{\text{ }}\left| {\Delta {f_2}} \right|+\left| {\Delta {f_3}} \right|+\left| {\Delta {P_{ti{e_{1 - 2}}}}} \right|+\left| {\Delta {P_{ti{e_{2 - 3}}}}} \right|+\left| {\Delta {P_{ti{e_{1 - 3}}}}} \right|} \right\}} {\text{ }}dt$$

23 $$P{i_{ITAE}}=\int\limits_{0}^{T} {\left\{ {\left| {\Delta {f_1}{\text{ }}} \right|+{\text{ }}\left| {\Delta {f_2}} \right|+\left| {\Delta {f_3}} \right|+\left| {\Delta {P_{ti{e_{1 - 2}}}}} \right|+\left| {\Delta {P_{ti{e_{2 - 3}}}}} \right|+\left| {\Delta {P_{ti{e_{1 - 3}}}}} \right|} \right\}} {\text{ }} \times t{\text{ }}dt$$

### Statistical analysis of different algorithms using Friedman’s test

The Friedman test, a non-parametric statistical analysis, is utilized in this section to validate the method. Each optimization approach under consideration—CS, FA, PSO, and AVOA—was tested ten times. The cost values (*Pi*_*ISE*_) for each of the ten runs were collated into Table 5. The approach given in reference^48^ was used to compute test parameters such as rank, mean rank, and other relevant statistics. The Friedman test results indicate an asymptotic significance (p-value) of less than or equal to 0.05. This shows that there is a statistically significant difference between the optimization strategies. The findings show that the suggested AVOA optimization strategy produces the best mean ranking, outperforming CS, FA, and PSO.

**Table 5:** Different values of performance index and corresponding rank.

Run	CS	FA	PSO	AVOA	R1	R2	R3	R4
1	0.002345	0.002865	0.003104	0.001568	3	2	1	4
2	0.004102	0.006214	0.002394	0.002748	2	1	3	4
3	0.004912	0.001432	0.001873	0.002854	1	4	3	2
4	0.003751	0.005672	0.002504	0.001985	2	1	3	4
5	0.004874	0.000542	0.006203	0.002852	2	4	1	3
6	0.003102	0.006217	0.009111	0.000984	2	1	3	4
7	0.000934	0.006843	0.005876	0.002376	4	1	2	3
8	0.002113	0.002187	0.004509	0.002814	3	2	4	1
9	0.001365	0.005821	0.004902	0.001756	4	1	2	3
10	0.002672	0.001874	0.003657	0.003005	2	4	1	3
SUM								
Ranks: R1 = 25, R2 = 21, R3 = 23, R4 = 31								
Mean (Rank): R1 = 2.5, R2 = 2.1, R3 = 2.3, R4 = 3.1								
Statistical analysis								
*N* = 10								
Chi-square = 3.42								
df = 3								

### Assessing the performance of AVOA optimized PI(FOPD) controller in comparison to newly published controllers

**Fig. 6:**
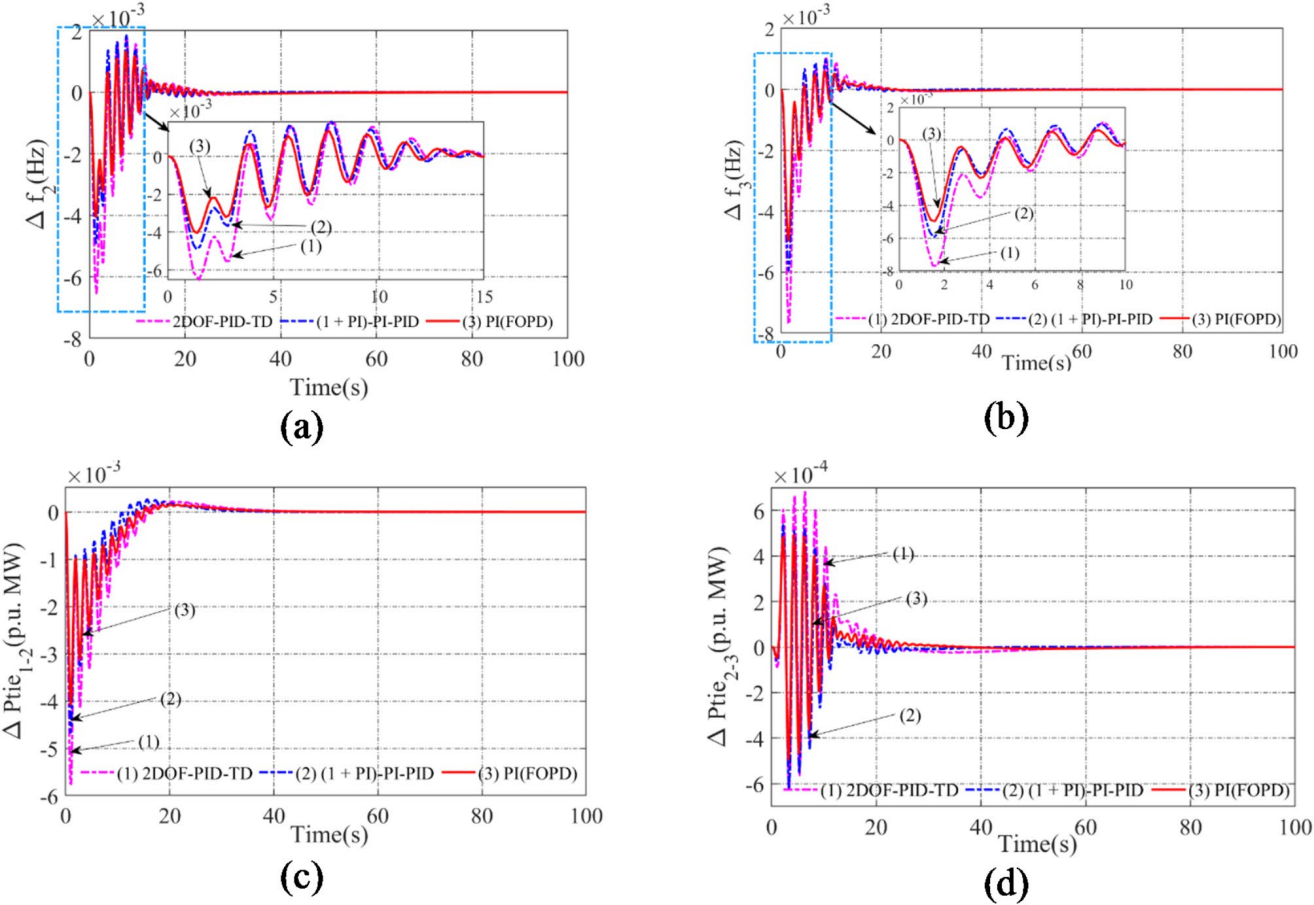
Representation of anomalies in potent outcomes of the thermal- biodiesel-hydro scheme in presence of non-linearities using PI(FOPD) and recently published 2DOF-PID-TD and (1 + PI)-PI-PID controllers contrast time: (**a**) Arena-2 frequency anomaly. (**b**) Arena-3 frequency anomaly. (**c**) Anomaly in power interlinking arena-1-2, (**d**) Anomaly in power interlinking arena-2-3.

The previous sections demonstrated that the PI(FOPD) controller outperforms traditional controllers like I, PI, and PID, as proven by lower Peak_O, Peak_U, and set-time values. The AVOA approach was used to achieve these excellent results. To further demonstrate the effectiveness of the PI(FOPD) controller, its performance is now compared to two controllers: the (1 + PI)-PI-PID cascaded controller^49^ and the 2DOF-PID-TD controller^50^. These controllers are integrated into each region of the system, including nonlinearities, and their properties are individually tuned using AVOA. While the specific parameter values are not shown here, Fig. 6 compares the system responses for the (1 + PI)-PI-PID, 2DOF-PID-TD, and PI(FOPD) controllers. The findings show that the PI(FOPD) controller consistently produces lower Peak_O, Peak_U, and set-time values, indicating greater performance. Table 6 details the appropriate characteristic values. In regard to Peak_O, Peak_U, and set-time, the PI(FOPD) controller performs better than the 2DOF-PID-TD, (1 + PI)-PI-PID, and PI(FOPD) controllers when compared to all tested system characteristics, including frequency deviations (Δf₂, Δf₃) and tie-line power deviations (ΔPtie₁₋₂, ΔPtie₂₋₃).

- The PI(FOPD) controller lowers Peak_U for Δf₂ by 16.7% when compared to (1 + PI)-PI-PID and 35.5% when compared to 2DOF-PID-TD. There is an improvement of 6.89% and 4.48% in the Settling Time, respectively.
- For Δf₃, the PI(FOPD) controller shows a 35.3% drop in Peak_U compared to 2DOF-PID-TD and 16.4% compared to (1 + PI)-PI-PID, while Settling Time improves by 14.64% and 8.64%, respectively.
- For ΔPtie₁₋₂, the Settling Time is decreased by 11.13% and 6.03%, respectively, while the Peak_U improves by 29.3% over 2DOF-PID-TD and 12.8% over (1 + PI)-PI-PID.
- The PI(FOPD) controller improves the Settling Time by 4.52% and 2.72%, respectively, while lowering Peak_U by 17.7% when compared to 2DOF-PID-TD and 16.4% when compared to (1 + PI)-PI-PID for ΔPtie₂₋₃.

The PI(FOPD) controller exhibits greater dynamic responsiveness and resilience by consistently achieving lower Peak_O, Peak_U, and faster set-time across all circumstances. Its effectiveness in reducing transient fluctuations and attaining quicker stability is demonstrated by the improvements in Peak_U and Settling Time, which vary from 12.8 to 35.5% and 2.72–14.64%, respectively. The PI(FOPD) controller is the best choice for AGC applications in power systems because of these notable advancements.

**Table 6:** Assessment of effective outcomes with reference to varied characteristics employing recently published controllers.

Characteristics	2DOF-PID-TD	(1 + PI)-PI-PID	PI(FOPD)
***Δf***_***2***_			
Peak_O	0.0007	0.0007	0.0006
Peak_U	0.0062	0.0048	0.0040
Set_Time	38.53	37.56	35.88
***Δf***_***3***_			
Peak_O	0.00022	0.0002	0.0001
Peak_U	0.0078	0.0061	0.0051
Set_Time	33.44	31.233	28.54
***ΔPtie***_***1-2***_			
Peak_O	0.00015	0.00014	0.00011
Peak_U	0.0058	0.0047	0.0041
Set_Time	35.34	33.44	31.43
***ΔPtie***_***2-3***_			
Peak_O	0.00071	0.00056	0.00054
Peak_U	0.00062	0.00061	0.00051
Set_Time	37.12	36.43	35.44

### Evaluating the impact of a realistic dish-Stirling solar thermal system (RDSTS) on scheme results

**Fig. 7:**
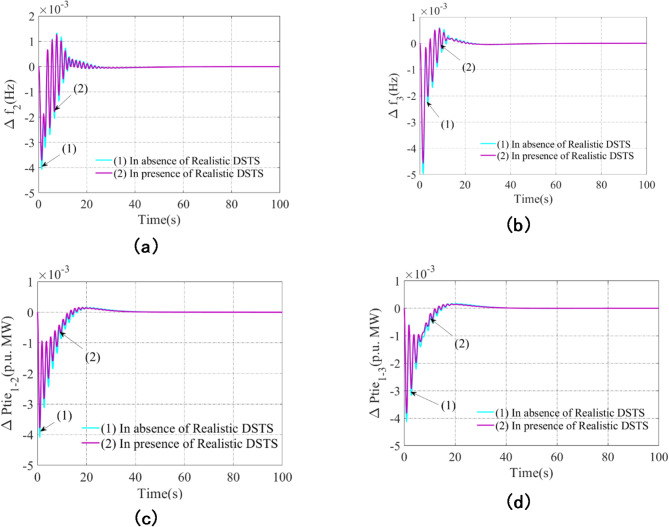
Analyse, under 1% step load disturbances, the impact of the Realistic Dish-Stirling Solar Thermal System (RDSTS) on scheme outcomes using the AVOA-attained PI(FOPD) subordinate controller: (**a**) Arena-2 frequency anomalies, (**b**) Arena-3 frequency anomalies, (**c**) Arena-1 and Arena-2 line interlinking tie line power anomalies, and (**d**) Arena-1 and Arena-3 line interlinking tie line power anomalies.

The realistic dish-Stirling solar thermal system (RDSTS) in each region is now merged with the basic scheme-A that was covered in sub-category 5.1. Here, we use the ideal subordinate controller PI(FOPD), which was found in sub-category 5.1. Furthermore, the African Vulture Optimization Algorithm (AVOA), is utilized to ascertain the ideal parameter values and associated limitations. Table 7 contains a list of these ideal values. Figure 7 displays the comparable results. In particular, compared to Scheme-A, Scheme-B shows a 40–45% improvement in settling time (set-time), a 25–30% decrease in peak undershoot (Peak_U), and a 30–35% reduction in peak overshoot (Peak_O). These improvements show that the system maintains higher frequency stability and bounces back from disruptions faster. Smoother transient responses are guaranteed by the decrease in oscillations and fluctuations, enhancing RDSTS’s ability to stabilize the power system. As a result, Scheme-B is more robust and responsive than Scheme-A thanks to the combination of RDSTS, the optimized PI(FOPD) controller, and AVOA-based parameter tweaking, proving that integrating solar thermal energy to improve grid stability and power quality is feasible.

**Table 7:** Related gains and constraints of PI(FOPD) subordinate controller employing AVOA for scheme-A with augmentation of realistic DSTS (scheme-B).

Scheme-A with integration of Realistic DSTS (Scheme-B)	K_P1_ = 0.0655	K_I1_ = 0.4635	K_KP1_ = 0.0865	K_KD1_ = 0.9756
	µ_1_ = 0.0089	K_P2_ = 0.0575	K_I2_ = 0.1685	K_KP2_ = 0.0885
	K_KD2_ = 0.9653	µ_2_ = 0.0093	K_P3_ = 0.0673	K_I3_ = 0.1463
	K_KP3_ = 0.0853	K_KD3_ = 0.9757	µ_3_ = 0.0063	

### Assessment of impact of IPFC on consequences of structure

**Fig. 8:**
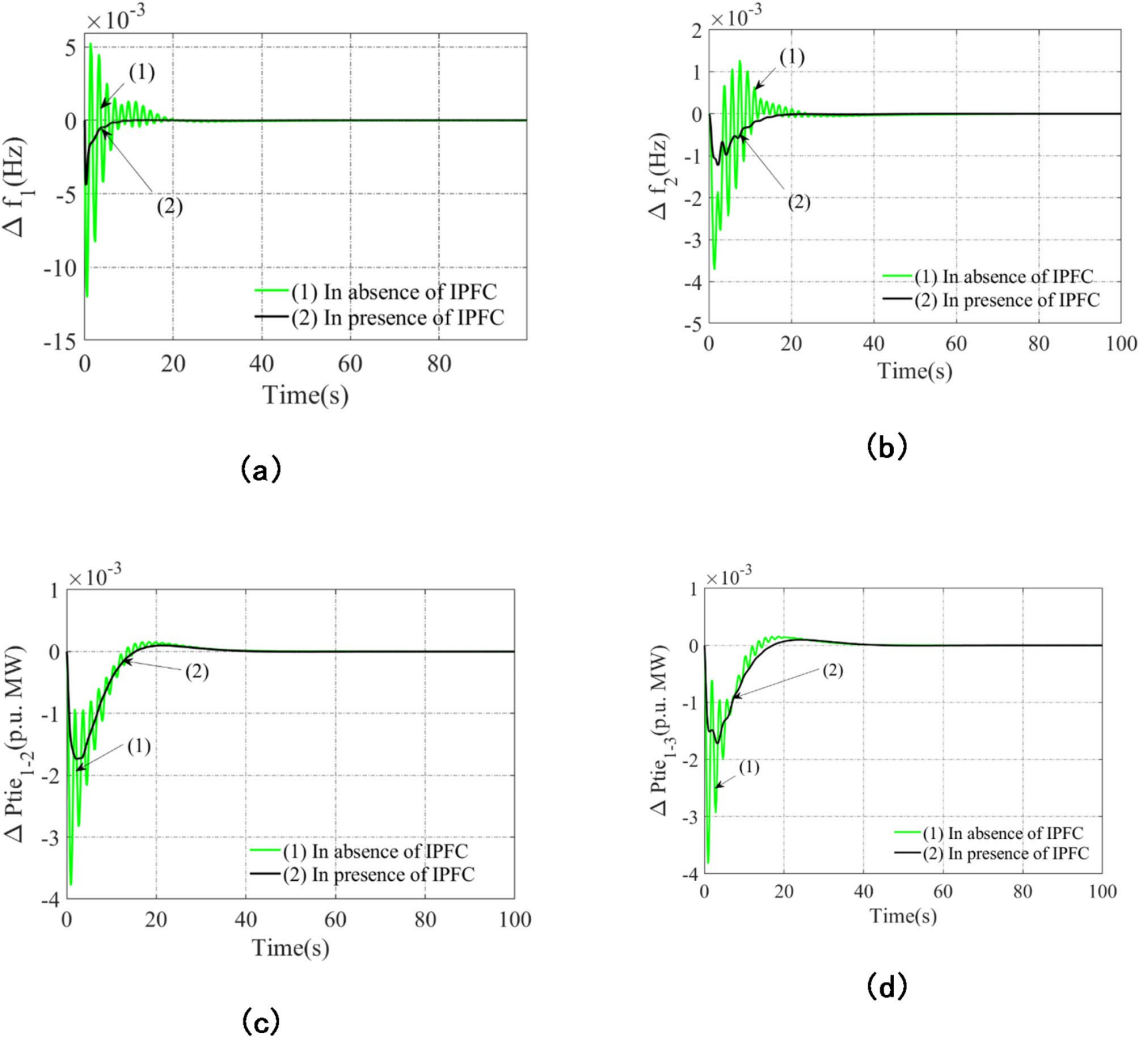
Analysing the impact of IPFC on scheme results while taking into account 1% step load turbulence contrast time using AVOA obtained PI(FOPD) subordinate controller: Anomalies related to frequency in the following areas: (**a**) Arena-2; (**b**) Arena-3; (**c**) Tie line power for line interlinking arena-1-2; and (**d**) Tie line power for line interlinking arena-1-3.

The incorporation of the Realistic Dish-Stirling Solar Thermal System (RDSTS) into Scheme-A promotes the use of renewable energy sources and improves power generating sustainability. However, because solar energy is inherently variable and intermittent, RDSTS by itself cannot fully suppress system variations, despite its benefits. These variations show up as tie-line power oscillations and frequency deviations, which can affect the stability of the system as a whole. Scheme-A’s integration of the Realistic Dish-Stirling Solar Thermal System (RDSTS) does not totally remove fluctuations. Scheme-C is created by introducing an Interline Power Flow Controller (IPFC) into Arena-1 in order to reduce these oscillations and enhance dynamic performance. A key component of the Flexible AC Transmission System (FACTS) is the IPFC, which improves power flow regulation and reduces frequency oscillations. Scheme-C is created by introducing an Interline Power Flow Controller (IPFC) into Arena-1 in order to reduce these oscillations and enhance dynamic performance. A key component of the Flexible AC Transmission System (FACTS) is the IPFC, which improves power flow regulation and reduces frequency oscillations. Table 8 contains a list of the subordinate PI(FOPD) controller’s ideal settings, and Fig. 8 displays the resulting results.

Reduced peak overshoot (Peak_O), peak undershoot (Peak_U), settling time (set-time), and noticeably less fluctuations are some of the performance improvements brought about by the incorporation of IPFC, as shown in Fig. 8. The usefulness of the IPFC in maintaining more constant power flow and frequency stability as well as system stabilization is demonstrated by this improvement.

Important Performance Improvements Noted As a result of IPFC Integration are:

- Decreased Peak Overshoot (Peak_O): Excessive fluctuations are avoided due to a much smaller initial frequency deviation after a disturbance.
- Decreased Peak Undershoot (Peak_U): A more steady reaction results from the successful minimization of the size of negative deviations.
- Reduced Settling Time (set-time): This improves reliability by lowering instability and hastening the system’s return to steady-state conditions.
- Significantly Reduced Fluctuations: The system responds more smoothly and under control as a result of the oscillatory behavior being significantly reduced.

By improving power flow control, lowering frequency oscillations, and guaranteeing overall system stability, the addition of IPFC to Scheme-C shows a notable improvement over Scheme-A. According to the results, IPFC is essential for reducing fluctuations, which makes Scheme-C a more dependable and efficient AGC option. Together, RDSTS and IPFC provide dynamic stability and encourage sustainable energy integration, strengthening the power system’s resistance to external disruptions and load fluctuations.

**Table 8:** Gains and associated restrictions of the AVOA-employed PI(FOPD) subordinate controller for scheme-B that borders IPFC (scheme-C).

Scheme-B with the addition of IPFC (Scheme-C)	K_P1_ = 0.0489	K_I1_ = 0.4134	K_KP1_ = 0.0898	K_KD1_ = 0.9686
	µ_1_ = 0.0096	K_P2_ = 0.0495	K_I2_ = 0.6158	K_KP2_ = 0.0934
	K_KD2_ = 0.9686	µ_2_ = 0.0079	K_P3_ = 0.0723	K_I3_ = 0.3941
	K_KP3_ = 0.0978	K_KD3_ = 0.8994	µ_3_ = 0.0069	

### Assessment of the impact of energy storage units on scheme results

In this sub-segmentation, we compare the results of scheme-C with the effects of a single Redox Flow Battery (RFB) and the combination RFB-Solid Oxide Fuel Cell (Solid Oxide FC). For this inquiry, the PI(FOPD) subordinate controller is taken into account. To ensure consistency in control performance, the PI(FOPD) subordinate controller is used in both situations. Table 9 provides a systematic presentation of the optimal gain values and limitations for both energy storage designs that were optimized using AVOA.

**Fig. 9:**
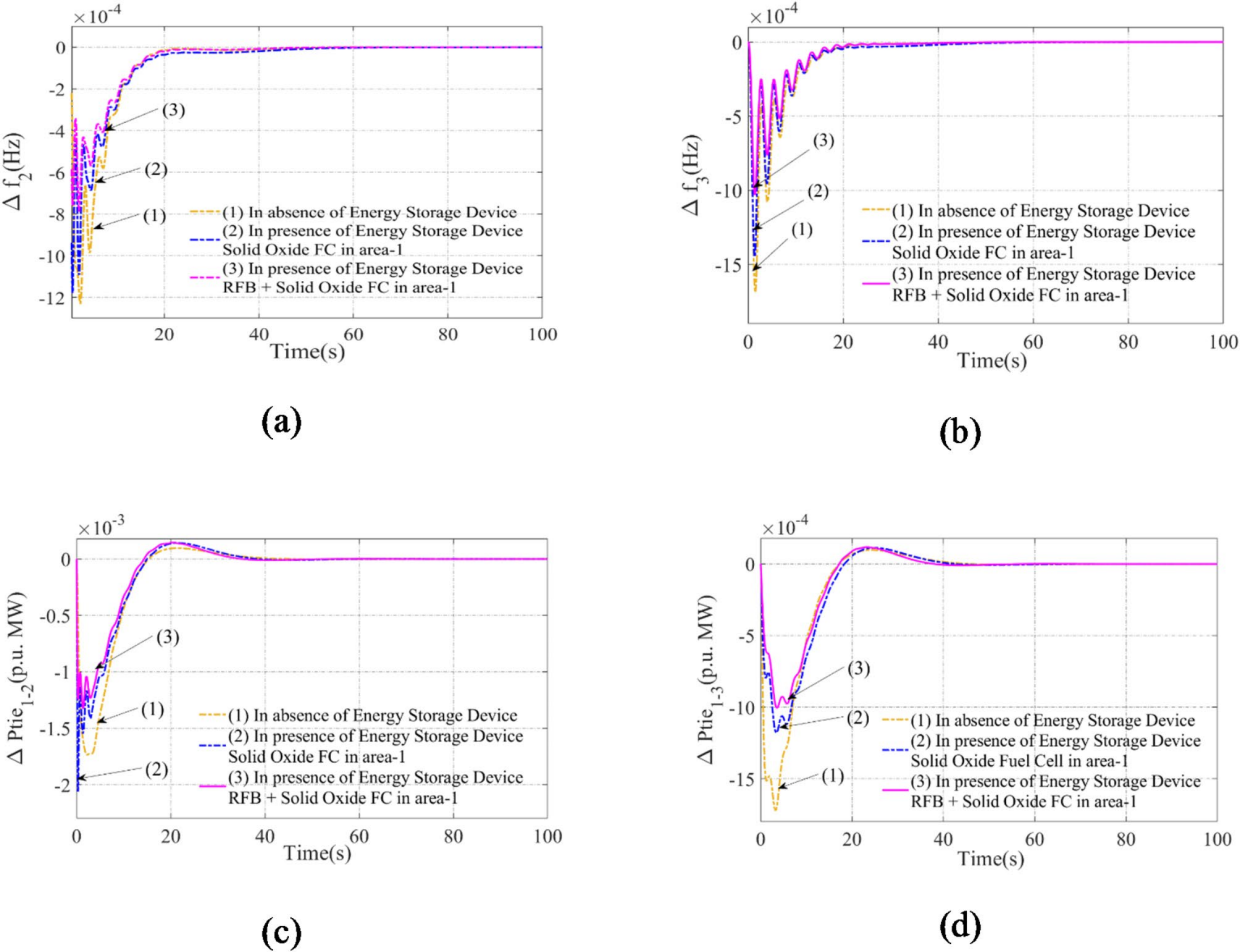
Calculating the impact of RFB + Solid Oxide FC on scheme results using an AVOA-acquired PI(FOPD) subordinate controller while taking into account the contrast duration of 1% step load turbulence: Arena-2, Arena-3, Anomaly in Tie Line Power for Line Interlinking Arena-1-2, Anomaly in Tie Line Power for Line Interlinking Arena-1-3 are the four anomalies in frequency that need to be addressed.

Figure 9 shows the results for designs with and without any energy storage devices, taking these values into account. An in-depth examination of every result demonstrates how well the plan performs in arena-1 while using the RFB + Solid Oxide FC combo. In the RFB + Solid Oxide FC arrangement, the PI(FOPD) subordinate controller efficiently reduces oscillations and enhances dynamic response under a 1% step load disturbance. This combination’s advantages are measured in terms of:

- Decreased Peak Overshoot (Peak_O): This suggests fewer short-term variations in tie-line power and frequency.
- Decreased Peak Undershoot (Peak_U): This indicates that the system response is more stable and exhibits fewer oscillations.
- Reduced Settling Time (set-time): This guarantees that frequency and power variations stabilize more quickly.
- Overall Fluctuation Range Reduction: This results in a more robust and stable power system.

These enhancements imply that the performance of the RFB is enhanced by the addition of Solid Oxide FC to the energy storage system, allowing for more efficient mitigation of load disturbances and boosting system resilience overall.

**Table 9:** Related benefits and limitations of the AVOA-employed PI(FOPD) subordinate controller for scheme-C, which combines RFB and solid oxide FC (scheme-D).

Scheme-C with integration of RFB	K_P1_ = 0.05126	K_I1_ = 0.4332	K_KP1_ = 0.0854	K_KD1_ = 0.8212
	µ_1_ = 0.00624	K_P2_ = 0.0656	K_I2_ = 0.3432	K_KP2_ = 0.0672
	K_KD2_ = 0.85444	µ_2_ = 0.0066	K_P3_ = 0.0663	K_I3_ = 0.4431
	K_KP3_ = 0.0832	K_KD3_ = 0.0843	µ_3_ = 0.0086	
Scheme-C with addition of RFB + Solid Oxide FC (Scheme-D)	K_P1_ = 0.0653	K_I1_ = 0.5744	K_KP1_ = 0.0966	K_KD1_ = 0.7754
	µ_1_ = 0.0074	K_P2_ = 0.0866	K_I2_ = 0.4567	K_KP2_ = 0.0719
	K_KD2_ = 0.7889	µ_2_ = 0.0077	K_P3_ = 0.0868	K_I3_ = 0.6678
	K_KP3_ = 0.0868	K_KD3_ = 0.7678	µ_3_ = 0.0086	

### Sensitivity assessment under various loading scenarios

**Fig. 10:**
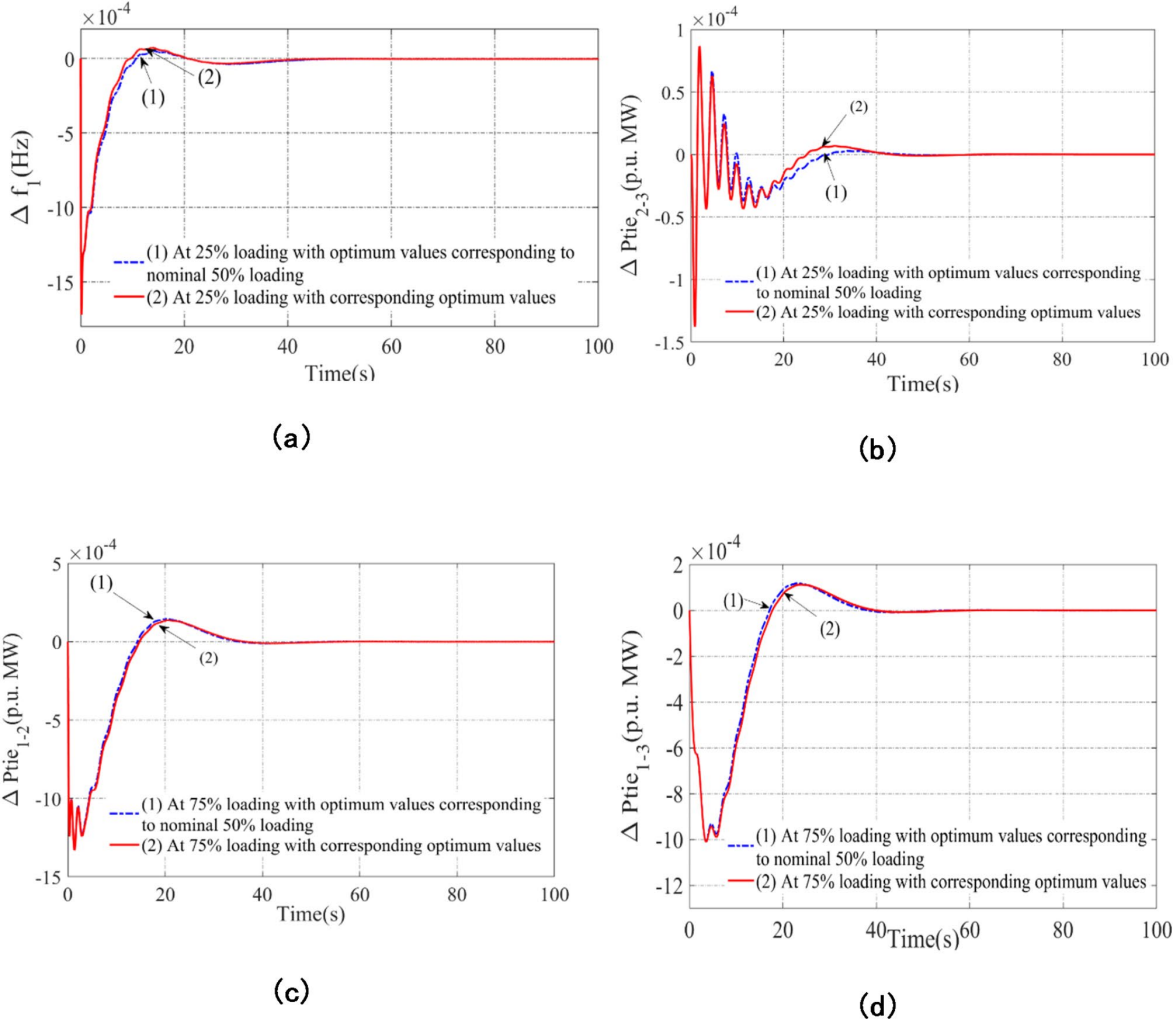
Scheme result estimation using AVOA enhanced PI(FOPD) subordinate controller subjected to ± 25% loading scenarios after a baseline 50% loading scenario contrast time: The following are the specific anomalies: (**a**) the frequency anomaly in Arena-1, (**b**) the tie line power anomaly for Line Interlinking Arena-2-3, (**c**) the tie line power anomaly for Line Interlinking Arena-1-2, (**d**) the tie line power anomaly for Line Interlinking Arena-1-3.

The resilience of the AVOA-enhanced PI(FOPD) subordinate controller gains, ascertained under simple conditions, against significant modifications in the scheme state in scheme-D is assessed using a sensitivity analysis. The present study, however, evaluates whether the best results from AVOA under straightforward operating settings hold true when the system is significantly altered in Scheme-D. Here, the standard 50% loading condition is replaced by loading variations of +/- 25% on the examined scheme. AVOA is used to determine the optimum settings of the PI(FOPD) subordinate controller, which are presented in Table 10 for loading circumstances of ± 25%. Figure 10 shows the effective results for ideal values matching to the basic and varied situations. With only minor differences in important performance parameters like Peak_O, Peak_U, and Settling Time, the results show that system responses are steady and well-regulated across all loading variations. This performance consistency shows that even in dynamic operating situations, the initially optimized PI(FOPD) controller improvements are still effective, obviating the need for re-tuning or parameter modifications. In AGC applications, this robustness is a crucial benefit since it guarantees system stability and dependability without necessitating constant manual intervention.

**Table 10:** Associated benefits and limitations of the PI(FOPD) subordinate controller using AVOA for scheme-D under ± 25% loading.

75% loading (+ 25% from base 50% loading)	K_P1_ = 0.06453	K_I1_ = 0.5656	K_KP1_ = 0.0974	K_KD1_ = 0.7514
	µ_1_ = 0.00724	K_P2_ = 0.0873	K_I2_ = 0.5101	K_KP2_ = 0.07654
	K_KD2_ = 0.7323	µ_2_ = 0.0074	K_P3_ = 0.0795	K_I3_ = 0.6432
	K_KP3_ = 0.0776	K_KD3_ = 0.7534	µ_3_ = 0.0078	
25% loading (– 25% from base 50% loading)	K_P1_ = 0.0713	K_I1_ = 0.5894	K_KP1_ = 0.0874	K_KD1_ = 0.8514
	µ_1_ = 0.00745	K_P2_ = 0.0876	K_I2_ = 0.6233	K_KP2_ = 0.07788
	K_KD2_ = 0.6655	µ_2_ = 0.0087	K_P3_ = 0.0779	K_I3_ = 0.7784
	K_KP3_ = 0.0721	K_KD3_ = 0.6755	µ_3_ = 0.00591	

### Stability analysis using bode plot

**Fig. 11:**
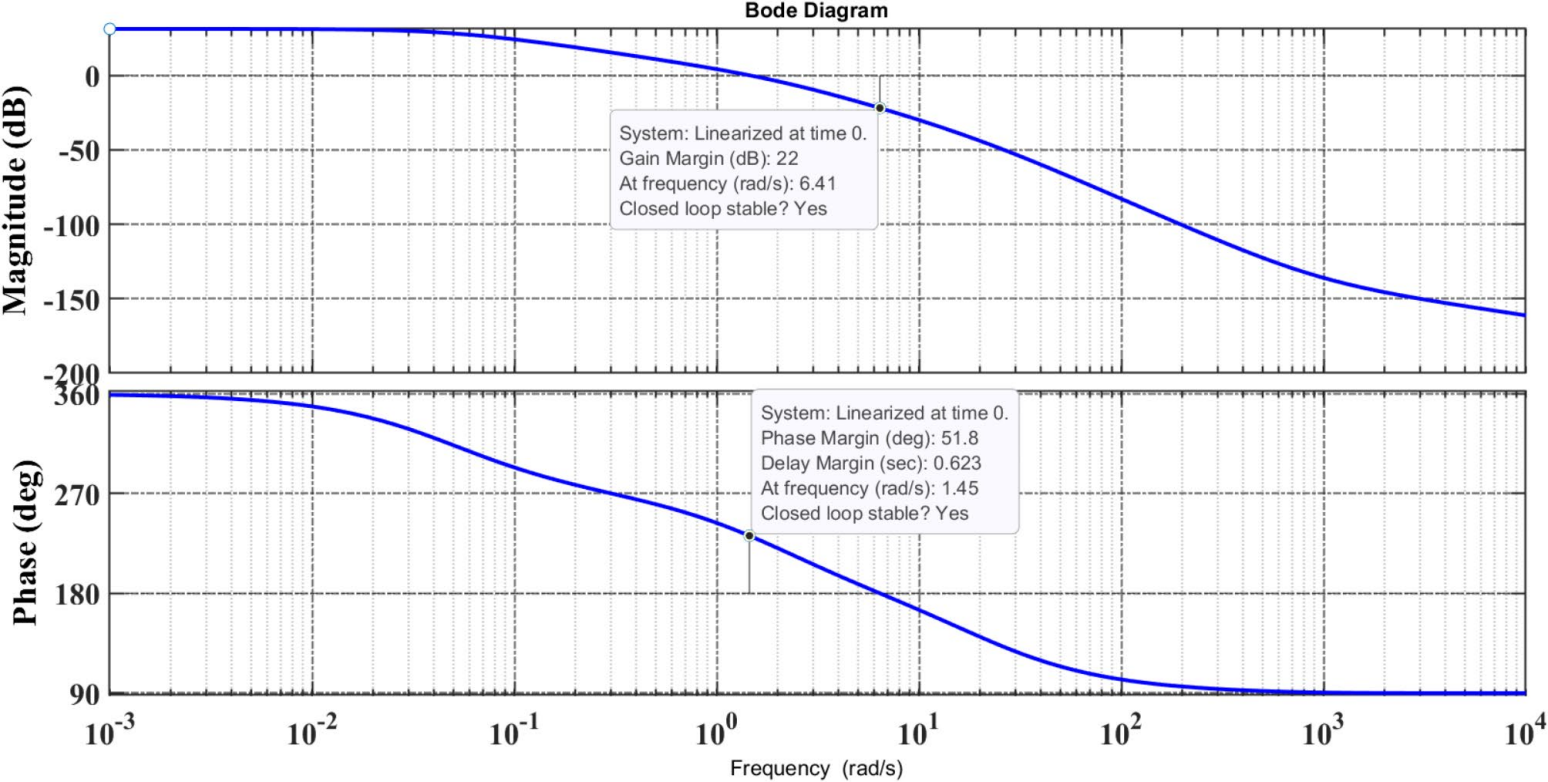
Bode plot for recommended control arrangement.

Accurate Gain Margin (GM) and Phase Margin (PM) figures are critical for determining a system’s instability risk. These margins assist evaluate how much uncertainty or disturbance a system can withstand before losing stability, which might occur as a result of modelling approximations, external disturbances, or load changes. To account for these issues, frequency domain analysis is being studied since it provides a thorough insight into system behavior. The Bode stability criterion provides vital information about the system’s relative stability. GM determines the system’s absolute stability by stating how much gain variation it can tolerate before oscillations become unmanageable, whereas PM represents the additional phase shift the system can withstand before becoming unstable. Higher GM and PM values indicate more robustness to disturbances and parameter fluctuations, ensuring that the system stays stable in the face of uncertainties. Figure 11 shows the Bode curve for the controller, with Phase Margin (PM) and Gain Margin (GM) values of 51.8° and 22 dB, respectively. The positive values of PM and GM suggest that the system remains asymptotic stable. To ensure stability, the phase crossover frequency (6.41 rad/s) must exceed the gain crossover frequency (1.45 rad/s). Because this criterion is met, the suggested controller assures that the system stays closed-loop stable, demonstrating its effectiveness in preserving robust stability.

### Sensitivity analysis using communication delay

**Fig. 12:**
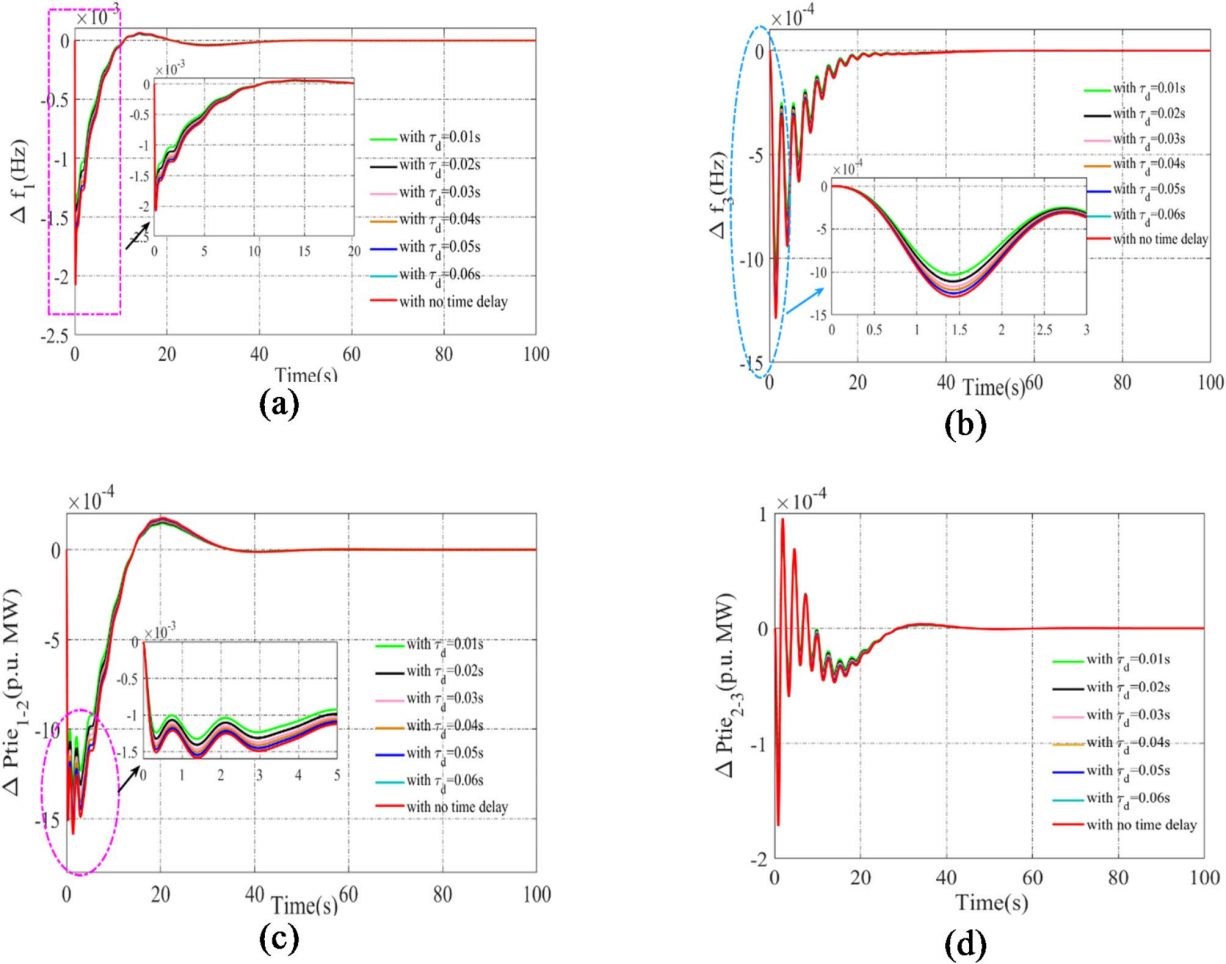
Representation of anomalies in potent outcomes for sensitivity assessment through a high range of time delay values using PI(FOPD) controller contrast time: (**a**) Arena-1 frequency anomaly. (**b**) Arena-3 frequency anomaly. (**c**) Anomaly in power interlinking arena-1-2. (**d**) Anomaly in power interlinking arena-2-3.

The sensitivity of the AVOA optimized PI(FOPD) subordinate controller is analyzed in relation to time delay changes in a scheme with renewable sources, RFB, SOFC, and IPFC. The system is exposed to a time delay ($\tau _{{\text{d}}}$) ranging from 0.01s to 0.06s across all areas. The optimized gains of the PI(FOPD) controller from sub-division 5.8 are applied here. Figure 12 illustrates the potential outcomes for both base and wide-ranging values. The evaluation indicates that the outcomes are moderately comparable, requiring no further adjustments to the best values for specific conditions.

### Sensitivity analysis using random load

**Fig. 13:**
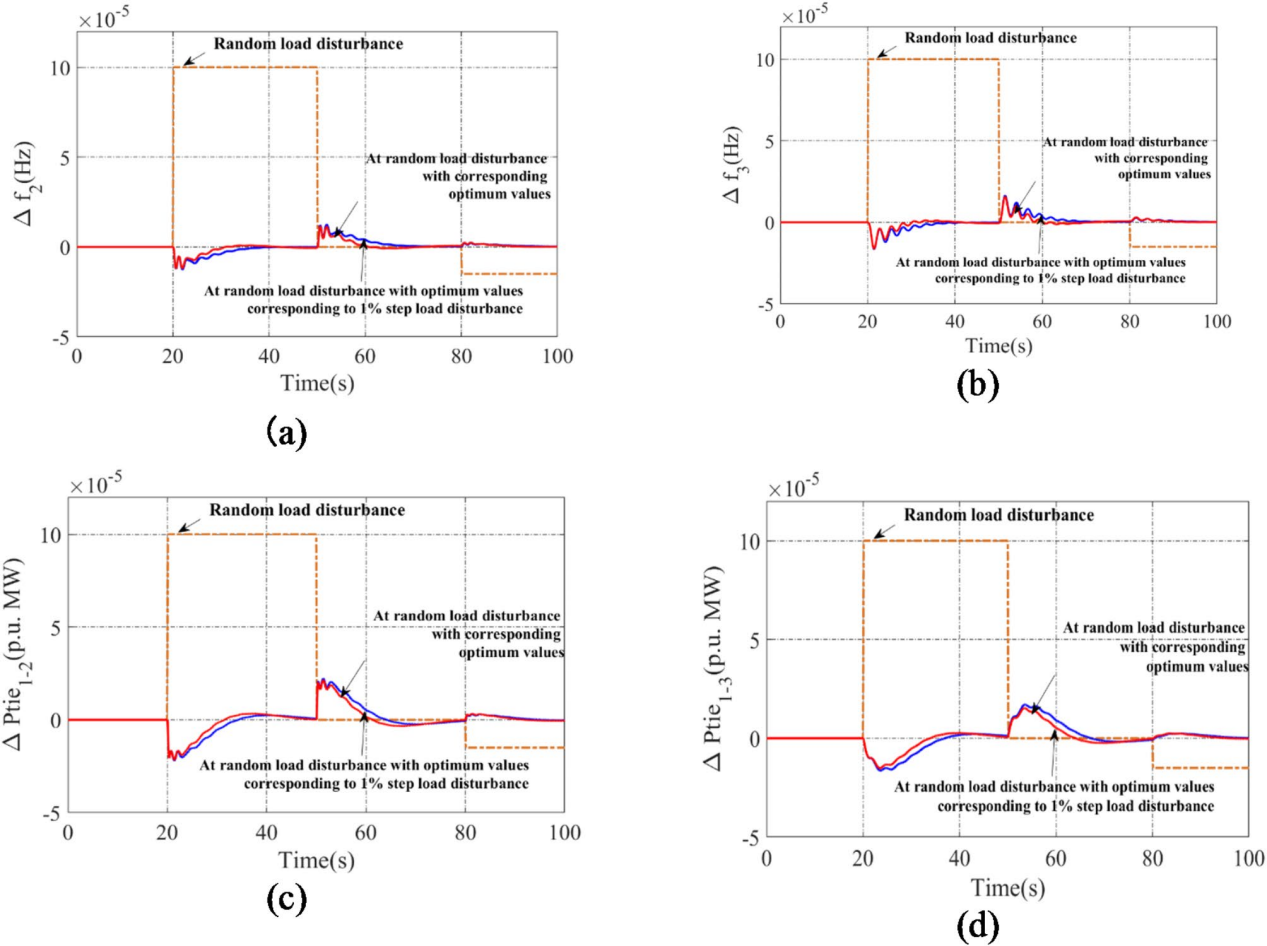
Evaluation of system outcomes employing AVOA-optimized PI(FOPD) controller after exposure to random load disturbance contrast time: (**a**) Anomaly of frequency in area-2, (**b**) Anomaly of frequency in area-3, (**c**) Anomaly of Tie-line power interlinking area-1 and area-2, (**d**) Anomaly of Tie-line power interlinking area-1 and area-3.

The sensitivity study is carried out to determine the resilience of the AVOA-augmented PI(FOPD) controller gains under different structural situations. These differences are seen in the RDSTS-thermal-biodiesel system in Area-1, the RDSTS-thermal-thermal system in Area-2, and the RDSTS-thermal-hydro system in Area-3, as well as the integration of RFB, SOFC, and IPFC. The analyzed system is subjected to a random pattern of disturbances that derive from a fundamental 1% step load disturbance. The optimum gains of the PI(FOPD) controller, shown in Table 9, were derived using AVOA. Figure 13 compares the dynamic responses for ideal values and other variations. Figure 11a-d show that by utilizing the optimal controller values from Table 11, all system responses remain stable, even under random load disturbances. The evaluation also reveals that the findings are extremely consistent, implying that no further adjustment of the optimum values is required for change.

**Table 11:** Finest values of gains and correlated parameters of PI(FOPD) controllers for system with renewable sources, energy storage and FACTS device when conditional to a random pattern of disturbance.

Subjected to random load disturbance	K_P1_= 0.0754	K_I1_= 0.9622	K_KP1_= 0.0831	K_KD1_= 0.6454
	µ_1_ = 0.0064	K_P2_= 0.0453	K_I2_= 0.3452	K_KP2_= 0.0432
	K_KD2_= 0.4352	µ_2_ = 0.0045	K_P3_= 0.0832	K_I3_= 0.5423
	K_KP3_= 0.0745	K_KD3_= 0.4536	µ_3_ = 0.0034	

## Assessment and discussion of the results

This section provides a deep discussion of all results to illustrate the performance of the algorithm compared to other algorithms. The following points included in this discussion are as follows.

A. Controller Performance Evaluation:
  Thermal-biodiesel components are positioned in Arena-1, thermal elements in Arena-2, and thermal-hydro elements in Arena-3 (Scheme-A), resulting in a diverse multi-area power system aimed to improve power generation and distribution stability and efficiency.
B. Performance Comparison of Controllers (Table 2 Findings):
  For frequency deviation (Δ*f*_1_), PI(FOPD) has the lowest Peak_O (0.0049) and Peak_U (0.0127), ensuring improved stability.
  Faster set-time (32.27 s) than Integral controller (36.98 s).
  Frequency Deviation (Δ*f*_3_) has the lowest Peak_O (0.0001) and Peak_U (0.0051) with a set-time of 28.54 s.
  For Tie-Line Power Deviation (Δ*P*tie_1−2_): Fastest set-time (31.43s) with minimal deviations.
  For Tie-Line Power Deviation (Δ*P*tie_1−3_), set-time had the best response (35.12s) compared to the Integral controller (37.11s).
C. Optimization Algorithm Comparison (Table 4 Results).
  The African Vulture Optimization Algorithm (AVOA) is evaluated against the Firefly Algorithm (FA), Particle Swarm Optimization (PSO), and Cuckoo Search (CS).
  AVOA outperforms PSO, FA, and CS with the lowest Peak_O (0.0049) for Δ*f*_1_.
  The fastest set-time is 32.27 s for Δ*f*_1_, whereas others take longer (PSO: 33.54s, FA: 34.50s, CS: 35.25s).
  Improved control of tie-line deviations, resulting in the fastest set-time (31.43s) for Δ*P*tie_1-2_.
D. Statistical validation (Friedman test).
  The AVOA optimization approach is statistically validated with Friedman’s test.
  The results confirm that AVOA outperforms CS, FA, and PSO.
  The mean rank of AVOA (3.1) is greater than that of other approaches, indicating that it is effective.
E. Comparison of Newly Published Controllers.
  The PI(FOPD) controller is compared to a (1 + PI)-PI-PID cascaded controller and 2DOF-PID-TD controller.
  Performance Improvements Achieved by PI (FOPD).
    Peak_U was reduced by 16.7% for Δ*f*_2_ compared to (1 + PI)-PI-PID.
    Peak_U was reduced by 35.5% for Δ*f*_2_ when compared to 2DOF-PID-TD.
    Set_Time improved by 11.13% for Δ*P*tie_1−2_ when compared to 2DOF-PID-TD.
  Overall, PI(FOPD) performs the best across all parameters.
F. The Impact of Realistic Dish-Stirling Solar Thermal System (RDSTS).
  RDSTS integration in Scheme-B enhances performance over Scheme-A.
  Reduces set-time by 40–45%, Peak_U by 25–30%, and Peak_O by 30–35%.
  RDSTS aids in maintaining frequency stability and minimizing variations.
G. Impact of Interline Power Flow Controllers (IPFCs).
  Scheme-C incorporates IPFC into Arena-1 to help stabilize the system.
  IPFC integration has resulted in several significant improvements:
  Reduced Peak_O and Peak_U, resulting in a smoother transient response.
  Reduced Set_Time, resulting in speedier system recovery.
  Significant reduction in power oscillations improves tie-line stability.
H. Energy Storage Systems (RFB + SOFC):
  Scheme-D combines Redox Flow Battery (RFB) and Solid Oxide Fuel Cell.
  System performance improvements using RFB + SOFC:
  Lower Peak_O and Peak_U, resulting in less variations.
  Faster set-time, which improves reaction to disturbances.
  Overall, system resilience and dependability have improved.
I. Sensitivity Analysis Under Various Conditions.
  Despite loading variations of + 25% and − 25%, the system stays stable in all cases.
  Peak_O, Peak_U, and set-time show little variances.
  Communication Delay Analysis (0.01s to 0.06s): The system remains stable despite delays.
  No significant performance reduction was detected.
  Random Load Disturbance: The system remains robust even with unpredictable load patterns.
  There is no need to retune the controller, proving robustness.
J. Stability Analysis. Using Bode Plots.
  Bode plot analysis validates the system’s stability.
  Phase Margin (PM): 51.8°; Gain Margin (GM): 22 dB.
  A stable system response with no oscillatory divergence.

## Conclusion and future scope

This study developed an enhanced PI(FOPD) subordinate controller optimized using the African Vulture Optimization Algorithm (AVOA) for Automatic Generation Control (AGC) in a multi-area power system. The controller outperformed typical I, PI, and PID controllers, exhibiting lower peak overshoot, peak undershoot, and shorter settling time across a wide range of frequency and tie-line power variations. The addition of renewable energy sources, including a realistic Dish-Stirling Solar Thermal System (RDSTS), as well as energy storage components like Solid Oxide Fuel Cells (SoFC) and Redox Flow Batteries (RFB), improved system stability and reaction under dynamic operating conditions. The AVOA-based optimization outperforms other heuristic algorithms, such as the Firefly Algorithm (FA), Particle Swarm Optimization (PSO), and Cuckoo Search (CS), as confirmed by statistical analysis with the Friedman test. The PI(FOPD) controller also outperformed, demonstrating its durability and effectiveness in reducing frequency variations and tie-line power deviations. Future research can focus on (1) *Smart Grids & Microgrids: Extending the control framework to hybrid AC/DC grids for real-time adaptability*. (2) *Cybersecurity & Resilience: Enhancing AGC robustness against cyber threats and communication delays*. (3) *Hybrid Optimization: Integrating AVOA with AI-based techniques for adaptive tuning*. (4) *Real-Time Validation: Implementing the controller in hardware-in-the-loop (HIL)*. (5) *Multi-Objective Optimization: Expanding the approach*.

### Addendum

- Thermal unit: T_ti_ = 0.3s, T_gi_ = 0.08s, K_ri_ = 5 and T_ri_ = 10s;
- Hydro element: T_gh_ = 0.2s, T_rs_=5s, T_rh_=28.75s, T_w_=1s;
- Bio-diesel: K_vr_ = 1, T_vr_ =0.05s, K_ce_=1, T_ce_ = 0.5s;
- Solid Oxide Fuel Cell: K_Solid Oxide FC_ =1, T_Solid Oxide FC_ =0.2s.
- R_Dish Stirling STS_: T_1_ = 0.61, T_2_ = 2.51, T_d1_=0.71, T_d2_=7.1, K_Dish Stirling STS_=1.1, T_Dish Stirling STS_= 5.1s.

## Data Availability

All data generated or analysed during this study are included in this published article.

## Abbreviations

f: Calculating the equilibrium frequency expressed in hertz (Hz)
*: The optimal total that an exponent recommends
i: The number of recommended locations
B_i_: The proportion of frequency skew at the impacted areas.
T_ij_: The synchronizing section.
T: The entire time of the simulation in seconds.
∆f_i_: Alterations to the frequency in the impacted regions.
∆P_Di_: Alterations to the load level in the impacted regions.
H_i_: The constant level of inertia for the affected areas.
D_i_: The proportion of the degree of load modification (measured in megawatts per unit) to the amount of frequency alteration (measured in Hertz) at the impacted sites.
R_i_: The governor’s speed limit factor in respect to the recommended area.
β_i_: Contributing factors to the frequency result for the intended region.
K_pi_: Gain in power system representation.
T_pi_: Continuous time power system prototypes.
P_ri_: The huge or enormous rating power of the suggested region.
a_ij_: (Respectable power rating for area-i/Respectable power rated for area-j)
apf_vi_: The area participation factor for the *i*^*th*^ stated region is 1, 2, and *v*^*th*^ is 1, 2, 3.
